# Deracemization of 1-phenylethanols in a one-pot process combining Mn-driven oxidation with enzymatic reduction utilizing a compartmentalization technique†

**DOI:** 10.1039/d2ra01326f

**Published:** 2022-04-06

**Authors:** Hirofumi Sato, Rei Yamada, Yomi Watanabe, Takaaki Kiryu, Shintaro Kawano, Motohiro Shizuma, Hideya Kawasaki

**Affiliations:** Osaka Research Institute of Industrial Science and Technology 1-6-50 Morinomiya, Joto-ku Osaka 536-8553 Japan hsato@omtri.or.jp; Kansai University 3-3-35 Yamatecho, Suita Osaka 564-8680 Japan

## Abstract

Racemic 1-phenylethanols were converted into enantiopure (*R*)-1-phenylethanols *via* a chemoenzymatic process in which manganese oxide driven oxidation was coupled with enzymatic biotransformation by compartmentalization of the reactions, although the two reactions conducted under mixed conditions are not compatible due to enzyme deactivation by Mn ions. Achiral 1-phenylethanol is oxidized to produce acetophenone in the interior chamber of a polydimethylsiloxane thimble. The acetophenone passes through the membrane into the exterior chamber where enantioselective biotransformation takes place to produce (*R*)-1-phenylethanol with an enantioselectivity of >99% ee and with 96% yield. The developed sequential reaction could be applied to the deracemization of a wide range of methyl- and chloro-substituted 1-phenylethanols (up to 93%, >99% ee). In addition, this method was applied to the selective hydroxylation of ethylbenzene to afford chiral 1-phenylethanol.

## Abstract 2

PDMS thimble was the key to combining incompatible reactions to achieve deracemization of 1-phenylethanols in high yield with high optical yield.

## Graphical abstract

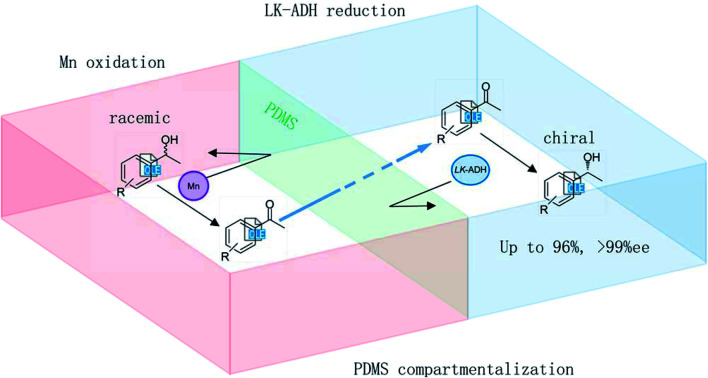

## Introduction

Optically-active secondary alcohols are the building blocks for a variety of pharmaceuticals, industrial chemicals, and natural products.^1–6^ Only the desired enantiomer is needed, and the antipode enantiomer is useless or just waste, which is a considerable economic burden.^7^ Hence, the synthesis of optically active compounds in organic chemistry has often been a complex multistep synthesis with repeated purification and isolation steps. Deracemization or optical inversion of the unwanted enantiomer is an ideal way to obtain the desired enantiomer.^8^ The study of deracemization to obtain optically pure secondary alcohols including optical inversion was first summarized by Carnell *via* dynamic kinetic resolution and the related deracemization concept.^9^ The field has been well systematized and much research has been reviewed in this decade.^10–14^

Recently, enzymatic oxidation/reduction has become a hot topic as a method to obtain optically pure secondary alcohols from unwanted “waste” enantiomers or inexpensive racemates.^15–17^ In particular, one-pot tandem reactions combining multiple enzymes have successfully overcome the weaknesses of multi-step synthesis, such as time wastage, isolation work that reduces the yield, and purification of intermediates.^8,18–21^ Moreover, the chemoenzymatic process, which combines organic and enzymatic reactions, is a very attractive process in that it benefits from both the wide substrate adaptation range of organic reactions and the stereospecificity of enzymes as well.^16,22–27^ Problems associated with the incompatibility of (bio)catalysts, however, are very frequent. The enzymes required for this process are those that are tolerant to organic catalysts and organic solvents and that do not lose their stereospecificity in their presence. This process has been also applied to the preparation of chiral secondary alcohol and efficient deracemization *via* dynamic kinetic resolution.^16,28–31^

A group of alcohol dehydrogenases, which are mostly NAD(P)H-dependent as cofactors, are enzymes that enantioselectively reduce ketone groups to produce optically active secondary alcohols, and due to their tolerance of organic solvents, they have been widely used in organic synthesis.^32–35^ ADH is often used in one-pot chemoenzymatic processes as well as in one-pot bioprocesses. However, ADH does not work well with some organic oxidants and metal catalysts due to its reducing properties and the fact that the cofactors used in combination with ADH are also vulnerable to oxidants.^22,23^ For example, when used in combination with TEMPO, a common mild oxidant, the sequence requires a quenching process prior to reduction by ADH.^36^ When used with a metal catalyst, it is necessary to select a reaction system that contains metals, ligands, and reagents that do not affect the reaction of ADH. Thus, the application of ADH to chemoenzymatic processes requires certain “tricks”.

To solve these problems, we have developed a compartmentalization method using a PDMS thimble.^37,38^ In this method, the copper(ii) ion of the Wacker catalyst, which is harmful to the enzyme, is sequestered in the interior chamber of a PDMS thimble, and only the products after the Wacker oxidation are transferred to the enzyme solution in the exterior chamber of the PDMS thimble, which allows the enzymatic reduction to be carried out efficiently. Using this system, we have achieved a one-pot process for the synthesis of optically pure 1-phenylethanol and 1-phenylethylamine from styrene. In the present study, we applied this process to the deracemization of secondary alcohol *via* a combination of oxidation using manganese oxide and asymmetric reduction using *LK*-ADH.^39,40^ A variety of chiral 1-phenylethanols were successfully prepared in a one-pot procedure in good yield and with excellent ee. Moreover, as a new approach without the use of optically active metal catalysts, the same process was utilized to synthesize enantiopure 1-phenylethanol from ethylbenzene.

## Results and discussion

### Optimized conditions of oxidation and reduction

Industrial applications of permanganate oxidant have been attracting a lot of attention because it is environmentally friendly. The oxidation of 1-phenylethanol was conducted according to the literature by Shaabani with slight modification.^41^ Racemic 1-phenylethanol was oxidized in the presence of Mn oxidant for 24 h at room temperature to afford acetophenone with 99% yield when 2 g mmol^−1^ (Mn oxidant/substrate) was used in methylene chloride (4 mL) (Table 1). The reaction proceeded quantitatively as long as there was no water, and no byproducts such as carboxylic acids or aldehydes were produced.

**Table 1:** Oxidation of 1-phenylethanol (1a) with Mn oxidant

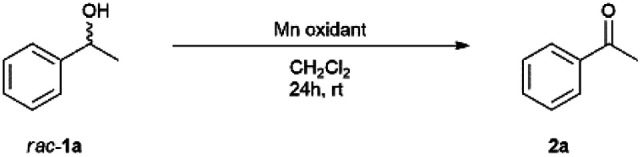			
*rac*-1a [mmol]	Mn oxidant [g]	CH_2_Cl_2_ [mL]	Yield^a^ [%]
1	2	4	>99

a Yield was determined by ^1^H-NMR.

The asymmetric reduction of acetophenone (2a) was conducted by an alcohol dehydrogenase from *Lactobacillus kefir* (*LK*-ADH) to afford (*R*)-1a with 96% yield and 99% ee under the optimized conditions with 120 U *LK*-ADH per mmol of the starting acetophenone (Table 2). The yield remained at 96% in the presence of 500 U *LK*-ADH per mmol due to 120 U *LK*-ADH per mmol being enough for the biotransformation of 2a to (*R*)-1a.

**Table 2:** Asymmetric enzymatic reduction of acetophenone (2a) by *LK*-ADH

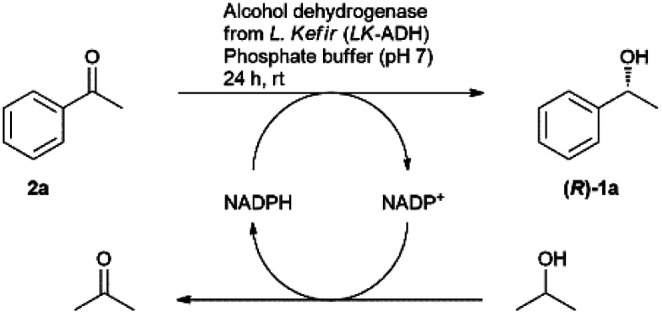				
Entry	2a [mmol]	Enzyme [U mmol^−1^]	Yield^a^ [%]	ee^b^ [%]
1	1	120	96	>99
2	1	500	96	>99

a Yield was determined by ^1^H-NMR.

b Ee was determined by chiral HPLC.

### Combining oxidation and reduction without a PDMS thimble

If the combination of Mn-driven oxidation and *LK*-ADH-driven reduction was compatible, the one-pot process of deracemization of secondary alcohol would directly proceed. However, in the initial attempt using a simple mixing system under optimized conditions (Table 3, entry 1), intermediate 2a was the only product with 95% yield. This indicated that the enzymatic reduction did not proceed or oxidation was dominant rather than enzymatic reduction in the system. The result was the same when an excess amount of enzyme (500 U mL^−1^) was used. Thus, the enzymatic reduction was possibly inhibited by the Mn species.

**Table 3:** Combining optimized conditions in a one-pot process

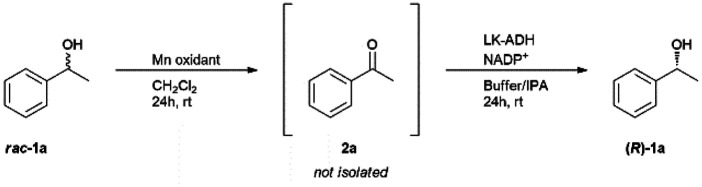				
Entry	*rac*-1a [mmol]	Enzyme [U mmol^−1^]	Yield^a^ [%]	
			2a	(*R*)-1a
1	1	120	96	n.d.^b^
2	1	500	96	n.d.^b^

a Yield was determined by ^1^H-NMR.

b n.d.: not detected.

To confirm this, the effect of MnO_2_ on the activity of *LK*-ADH was investigated. The activity before MnO_2_ addition was normalized to 1.0 and the activity after addition of MnO_2_ was compared with the original (Fig. S1†). The activity immediately decreased to half by adding MnO_2_ and was lost after 60 min.

*LK*-ADH needs a cofactor, NADP^+^ or NADPH, to reduce ketone 2a to obtain (*R*)-1a. To be able to use this cofactor in catalytic amounts, the oxidized cofactor is continuously reduced by IPA. Because the oxidation of cofactor NADPH by Mn oxidant might also cause deactivation, the UV profile was measured in the presence and absence of MnO_2_. The typical absorption at 340 nm decreased with the addition of MnO_2_ into the NADPH solution, indicating that NADPH was oxidized. These results showed that the Mn oxidant has a significantly negative impact on both enzyme and cofactor, and stopped the sequential reaction halfway.

### Sequential deracemization of *rac*-1a by using a PDMS thimble

**Fig. 1:**
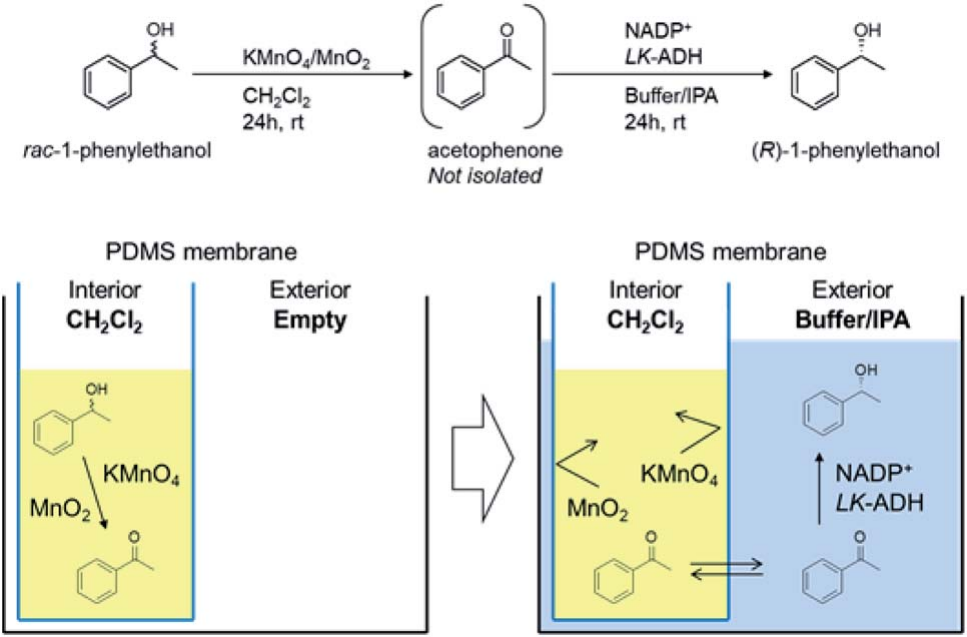
Concept of confinement of the Mn oxidant by using a PDMS thimble toward the combination of Mn-driven oxidation and enzymatic reduction. Oxidation was conducted in the PDMS interior chamber, then enzymatic reduction was conducted in the exterior chamber sequentially.

In order to achieve the one-pot deracemization of 1-phenylethanol by a combination of Mn-driven oxidation and enzymatic reduction by *LK*-ADH, we applied the compartmentalization technique with a PDMS membrane.^37,38^ Hydrophilic compounds such as Mn salt and enzyme are not able to pass bi-directionally through the hydrophobic PDMS membrane, so the enzyme might be protected from the Mn oxidant (see ESI†). Due to the hydrophobicity of the membrane, 2a formed *in situ* in the interior chamber could flux the membrane and diffuse into the exterior chamber to be converted into (*R*)-1a in the presence of the enzyme. The deracemization process using a PDMS thimble is illustrated in Fig. 1. The PDMS thimble was settled in a flask. Mn-driven oxidation of *rac*-1a was conducted in the interior of the PDMS thimble, and subsequently phosphate buffer, IPA, NADP^+^, and *LK*-ADH were added into the exterior to flux the generated 2a. In the exterior chamber, 2a was converted into chiral (*R*)-1a.

Adopting the optimized conditions of Mn-driven oxidation in the interior chamber and enzymatic reduction by *LK*-ADH in the exterior chamber, which was described in the former section, (*R*)-1a was obtained at 31% chemical yield and 99% ee in the exterior liqueur, indicating that the one-pot process was actually achieved by using a PDMS thimble (Table 4, entry 1). Here, it was found that 19% of 2a and 4% of 1a remained in the interior liquor (Table 4, entry 1). Thus, the volume of IPA in the exterior liquor was increased to incite the compound's diffusion (entry 2). As a result, neither 2a nor 1a was detected in the interior chamber by the IPA increment. However, the reduction by *LK*-ADH was still incomplete, retaining the content of intermediate 2a at 35%. When the activity of *LK*-ADH was increased to 500 U mmol^−1^ to promote the reduction, the yield of (*R*)-1a was improved to 86% (Table 4, entry 3). To further improve the yield of (*R*)-1a, the amount of Mn oxidant was increased (Table 4, entries 4–7) from 0.5 g to 3.0 g. The yield of (*R*)-1a, however, was decreased and the remaining amount of 2a was increased. This result implies that the excessive use of an Mn oxidant induced the re-oxidation of (*R*)-1a. In the case of 0.5 g of Mn oxidant, the optical yield decreased to 64% ee (Table 4, entry 4), probably due to insufficient oxidation. Taking these results into consideration, the conditions shown in Table 4, entry 5 (yield of 96% and optical yield of 99% ee) were revealed to be optimal.

**Table 4:** Optimization of a sequential one-pot process with PDMS thimble

Entry	Chamber	*rac*-1a [mmol]	Mn oxidant [g]	CH_2_Cl_2_ [mL]	Enzyme [U mmol^−1^]	NADP^+^ [μmol]	Buffer [mL]	IPA [mL]	Yield^a^ [%] (ee^b^)	
									2a	(*R*)-1a
1	Interior	1	2	4	—	—	—	—	19	4 (93% ee)
	Exterior	—	—	—	120	20	15	5	42	31 (>99% ee)
2	Interior	1	2	4	—	—	—	—	n.d.^c^	n.d.^c^
	Exterior	—	—	—	120	20	15	15	35	57 (>99% ee)
3	Interior	1	2	4	—	—	—	—	n.d.^c^	n.d.^c^
	Exterior	—	—	—	500	20	15	15	13	86 (>99% ee)
4	Interior	1	0.5	4	—	—	—	—	n.d.^c^	n.d.^c^
	Exterior	—	—	—	500	20	15	15	3	95 (>64% ee)
5	Interior	1	1	4	—	—	—	—	n.d.^c^	n.d.^c^
	Exterior	—	—	—	500	20	15	15	4	96 (>99% ee)
6	Interior	1	1.5	4	—	—	—	—	n.d.^c^	n.d.^c^
	Exterior	—	—	—	500	20	15	15	6	91 (>99% ee)
7	Interior	1	3	4	—	—	—	—	n.d.^c^	n.d.^c^
	Exterior	—	—	—	500	20	15	15	14	84 (>99% ee)

a Yield was calculated from ^1^H-NMR.

b Enantiomeric excess of (*R*)-1-phenylethanol was determined by chiral HPLC.

c n.d.: not detected.

Applying these optimized conditions, chiral (*S*)-1a was converted into the antipode (*R*)-1a in 96% yield, 99% ee optical yield, indicating that a one-pot stereo inversion was successfully achieved by the compartmentalization technique.

The optimized method uses a stepwise method, where the oxidation in the interior chamber is done first, followed by the exterior chamber. While it is tempting to perform these reactions simultaneously, the efficiency of the reaction was insufficient because the solvent in the interior chamber diffused into the exterior chamber before the oxidation was complete.

The combination of permanganate and dichloromethane may form phosgene. Isooctane was considered instead of dichloromethane as an interior solvent. Although the oxidation reaction proceeded quantitatively, the diffusion of the acetophenone produced in the interior chamber to the exterior chamber was not sufficient under the conditions of this experiment, resulting in dichloromethane being the most suitable solvent.

### Substrate scope

Based on the optimal conditions for the one-pot production of chiral (*R*)-1a, this methodology was extended to the deracemization of a series of aromatic racemic alcohols (Table 5 and ESI†). Deracemization of substituted 1 proceeded at relatively high optical yield, but the chemical yield was insufficient in the case of *ortho*- and *meta*-substituted substrates. Taking the sum of the corresponding acetophenone yield and 1-phenylethanol yield into consideration, the lower chemical yield in the case of an *ortho*-substituted substrate might be responsible for the substrate specificity of *LK*-ADH derived from steric hindrance (Table 5, entries 1 and 4).^37,40^ Substrate chloroacetophenones mostly showed better yield than methyl acetophenones. This may be because the enzymatic reduction was accelerated by an electron-withdrawing group which stabilizes the negative charge on the substrate and helps hydride-transfer from NADPH to a substrate by an electron–proton–electron multistep mechanism.^45,46^

**Table 5:** Substrate scope of the deracemization method^a^

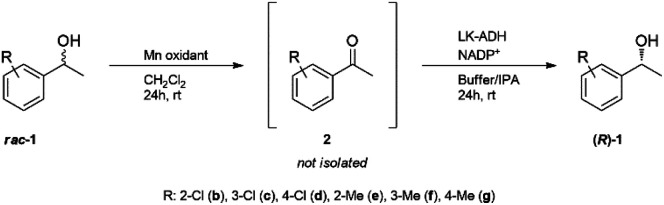				
Entry	Substrate	Chamber	Yield^b^ [%] (ee^c^)	
			2	(*R*)-1
1	2-Cl	Interior	n.d.^d^	n.d.^d^
	(1b)	Exterior	63	32 (>91% ee)
2	3-Cl	Interior	n.d.^d^	n.d.^d^
	(1c)	Exterior	9	83 (>99% ee)
3	4-Cl	Interior	n.d.^d^	n.d.^d^
	(1d)	Exterior	2	93 (>99% ee)
4	2-Me	Interior	n.d.^d^	n.d.^d^
	(1e)	Exterior	87	5 (86% ee)
5	3-Me	Interior	n.d.^d^	n.d.^d^
	(1f)	Exterior	29	65 (>99% ee)
6	4-Me	Interior	n.d.^d^	n.d.^d^
	(1g)	Exterior	11	82 (>99% ee)

a Mn oxidant (1 g) was added to 250 mM of *rac*-1-phenylethanols/4 mL of CH_2_Cl_2_ in the PDMS interior chamber. Buffer (15 mL, pH 7), IPA (15 mL), NADP^+^ (20 μmol) and *LK*-ADH (500 U mmol^−1^) were added to the exterior chamber.

b Yield was calculated from NMR.

c Enantiomeric excess of (*R*)-1-phenylethanols was determined by chiral HPLC.

d n.d.: not detected.

### Selective hydroxidation of ethylbenzene

The selective hydroxidation of hydrocarbons is another key research area in academia and industry.^47^ In the reaction, optically active secondary alcohols are often synthesized using optically active metal complexes, but it is not easy to obtain the absolute enantiomer.^48^ Thus, an enzymatic process has been applied to the field these days.^49^ Our method also has the advantage of using a biocatalyst to obtain the product with absolute enantiomeric excess. We applied this one-pot methodology to the conversion of ethylbenzene into chiral 1-phenylethanol (Table 6). The first trial, which was conducted under the same conditions as 1-phenylethanol deracemization, afforded 17% (*R*)-1a and 53% of the substrate ethylbenzene (3) remained (Table 6, entry 1). The conversion was lower compared to the deracemization of phenylethanols. The intermediate acetophenone both in the interior and the exterior was completely consumed, suggesting that enzymatic reduction had completely proceeded. When more Mn oxidant was added to the interior, the yield of (*R*)-1a improved to 42% (Table 6, entry 2). As the capacity of the PDMS thimble we had prepared this time allowed a maximum of 3 g of Mn oxidant, we could not increase the amount to more than 3 g. Therefore, a chemical yield of 42% and optical yield up to 99% was the limit of the experiments this time.

**Table 6:** Selective hydroxylation of ethylbenzene

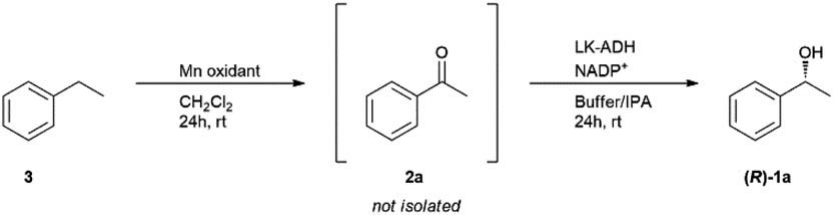					
Entry	Chamber	Mn oxidant [g]	Yield^a^ [%] (ee^b^)		
			3	2	(*R*)-1a
1	Interior	1	6	n.d.^c^	n.d.^c^
	Exterior	—	53	1	17 (>99% ee)
2	Interior	3	n.d.^c^	n.d.^c^	n.d.^c^
	Exterior	—	28	3	42 (>99% ee)

a Yield was calculated by HPLC.

b Enantiomeric excess was determined by chiral HPLC.

c n.d.; not detected.

## Experimental

### General

All reagents were purchased from FUJIFILM Wako Pure Chemical Corporation, Nacalai Tesque, Inc., Kanto Chemical Co., Inc., Sigma-Aldrich Co. LLC., and Tokyo Chemical Industry Co., Ltd. The reagents were used without further purification. Coenzyme NADP^+^ and NADPH were purchased from Oriental Yeast Co., Ltd. ^1^H-NMR and ^13^C-NMR were recorded on a JEOL ECA-600 or a JEOL AL-300 spectrometer. Chemical shifts were reported in ppm relative to TMS, *δ* (^1^H) = 0.00 ppm, *δ* (^13^C) = 0.00 ppm. Enantiomeric excesses were determined by HPLC analysis equipped with a chiral column and a mixture of i-PrOH/*n*-hexane as the mobile phase at a flow rate of 1 mL min^−1^ at 25 °C (see ESI†). HPLC was performed on a combined system: Shimadzu SCL-10AVP system controller, SIL-10ADVP auto sampler, LC-10ADVP high pressure pump, DGU-14A degasser, SPD-10AVVP UV detector, and CTO-10ACVP column oven. Photometer assays were conducted on a JASCO UV-560 or a JASCO V-780 at 340 nm. The alcohol dehydrogenase from *Lactobacillus kefir* (*LK*-ADH^39,40^) was used as a crude extract, which was diluted with glycerin in a 1 : 1 ratio and stored in a freezer until use. The volume-activity of this solution was determined by photometer-assay shortly prior to use. Unless otherwise stated, a phosphate buffer of pH 7.0 prepared from NaH_2_PO_4_/K_2_HPO_4_ at a concentration of 50 mM was used as a buffer. Analytical thin layer chromatography was performed with a Merck silica gel 60 F254 25 glass plate, layer thickness 0.2 mm. Components were visualized by UV or anisaldehyde/sulfuric acid staining or iodine staining. Open column chromatography purification was performed in 0.063–0.2 mm neutralized silica gel purchased from Kanto Chemical Co., Inc. The PDMS thimble was fabricated by the method previously reported.^37^

### Preparation of the Mn oxidant (MnO_2_/KMnO_4_)

The Mn oxidant (MnO_2_/KMnO_4_) was prepared according to the literature report by Shabaani with slight modification.^41^ KMnO_4_ (0.5 g, 3.2 mmol) and MnO_2_ (1.5 g, 17.3 mmol) were crushed with a mortar until they became a uniform powder to prepare fresh Mn(iv) species. The Mn oxidant was prepared shortly prior to use.

### Membrane permeability of Mn measured by ICP-AES^42–44^

The Mn oxidant (KMnO_4_/MnO_2_, 1 : 3 w/w) and a magnetic stirrer bar were placed in a PDMS thimble. The PDMS thimble was placed in a round-bottomed flask and dichloromethane (4 mL) was added to the inside of the PDMS thimble. Subsequently Milli-Q water (15 mL), IPA (15 mL), and a magnetic stirrer bar were added to the exterior of the PDMS thimble. The mixture was stirred for 24 hours at room temperature. The aqueous phase was evaporated to concentrate it and fixed at 30 mL with additional Milli-Q water, and then sulfuric acid (98%, 30 μL) was added to the solution. A measurement sample was prepared from the solution after filtration with a membrane filter. The PDMS membrane permeability for Mn was evaluated with a Thermo Fisher SCIENTIFIC iCAP-7400 (exposure time: 15 s, RF power: 1150 W, nebulizer gas flow: 0.5 L min^−1^, coolant gas flow: 12 L min^−1^, support gas flow: 0.5 L min^−1^, measurement mode: axial). The amount of added Mn oxidant was 0 g (zero control), 0.5 g, 2.0 g, and 3.0 g, respectively, for each experiment (ESI†).

### General method for the combination of Mn-driven oxidation of 1-phenylethanol in the interior and enzymatic reduction in the exterior of the PDMS thimble

A magnetic stirrer bar was placed in a PDMS thimble, and then the PDMS thimble was placed in a round-bottomed flask. A dichloromethane solution of *rac*-1-phenylethanol (*rac*-1a, 122 mg, 1.00 mmol in 4.0 mL) was added to the inside of the PDMS thimble, and then Mn oxidant was also added to the inside of the PDMS thimble. The mixture was stirred for 24 hours at 25 °C. After completion of the oxidation, i-PrOH (15 mL), phosphate buffer solution (15 mL, 50 mM, pH 7.0), and another magnetic stirrer bar were added to the exterior of the PDMS thimble. Then, NADP^+^ (15 mg, 20 μmol) and ADH from *Lactobacillus kefir* (*LK*-ADH, 500 U mmol^−1^, 1400 U mL^−1^) were added to the exterior and the reaction mixture was stirred for 24 hours at 25 °C. After the reaction, the PDMS thimble was removed from the flask, and then the aqueous phase in the flask was subsequently extracted with chloroform (10 mL × 3), and the combined extracts were dried over sodium sulfate and concentrated in a vacuum. To quantify and qualify the product in the interior, the removed PDMS thimble with Mn oxidant debris was also washed with chloroform (10 mL × 3), and then filtered. The filtrate was concentrated under vacuum. The amount of resulting products both from the interior and the exterior were quantified by ^1^H-NMR with *t*-BuOH as an internal standard. The enantiomeric excess was evaluated by chiral HPLC. The amount and the volume of each reagent were changed in each experiment.

### Effect of Mn oxidant on the activity of *LK*-ADH

The activity of *LK*-ADH solution was measured with a JASCO V-560 UV-vis spectrometer (response: medium, bandwidth: 2.0 nm, monitoring wavelength: 340 nm, time course: 60 s, temperature: 25 °C). An enzyme solution of which the initial activity was 20 U mL^−1^ was prepared, and then MnO_2_ (50 mg) was added to the solution. The mixture was stored at 25 °C. The activity was measured immediately, 10 min, and 60 min after the addition. The concentrations of acetophenone substrate and NADPH cofactor were 10 mM and 6.25 mM, respectively.

## Conclusions

The use of an Mn oxidant in the interior of a PDMS thimble enabled us to combine the reaction with an enzymatic reduction in a one-pot process although MnO_2_ is highly detrimental to the enzyme. In the one-pot process, as the generated intermediate acetophenone in the interior chamber is automatically extracted by the medium of the sequential enzymatic reduction in the exterior chamber, an isolation step which takes time and effort can be avoided. By the one-pot process, not only achiral non-substituted 1-phenylethanol but methyl- and chloro-1-phenylethanols were successfully converted into the corresponding chiral forms with high yield and excellent enantioselectivity except for the *ortho*-isomer. Moreover, selective hydroxidation of ethylbenzene into chiral 1-phenylethylethanol was achieved by applying this method.

## Author contributions

The study was conceptualized by HS. Experiments were conducted by RY with initial support by YW, TK, MS and HK. The manuscript was written by HS and checked by all the co-authors.

## Conflicts of interest

There are no conflicts to declare.

## Supplementary Material

RA-012-D2RA01326F-s001

## References

[cit1] Patel R. N. (2013). Biomolecules.

[cit2] Patel R. N. (2008). Coord. Chem. Rev..

[cit3] Arai N. and Ohkuma T., Reduction of Carbonyl Groups: Hydrogenation, ed. G. A. Molander, Thieme, New York, 2011, vol. 2.1, p. 9

[cit4] Breuer M., Ditrich K., Habicher T., Hauer B., Keseler M., Stürmer R., Zelinski T. (2004). Angew. Chem., Int. Ed..

[cit5] Li Z., Yang H., Liu J., Huang Z., Chen F. (2021). Chem. Rec..

[cit6] Ohkuma T. (2010). Proc. Jpn. Acad., Ser. B.

[cit7] Gruber C. C., Lavandera I., Faber K., Kroutil W. (2006). Adv. Synth. Catal..

[cit8] Voss C. V., Gruber C. C., Faber K., Knaus T., Macheroux P., Kroutil W. (2008). J. Am. Chem. Soc..

[cit9] Carnell A. J. (1999). Adv. Biochem. Eng./Biotechnol..

[cit10] Ramano D., Villa R., Molinari F. (2012). ChemCatChem.

[cit11] Musa M. M., Vieille C., Phillips R. S. (2021). ChemBioChem.

[cit12] Ahmad I., Shagufta A. M., Abdul R. (2017). Chirality.

[cit13] Pellissier H. (2018). Tetrahedron.

[cit14] Musa M. M., Hollmann F., Mutti F. G. (2019). Catal. Sci. Technol..

[cit15] Voss C. V., Gruber C. C., Kroutil W. (2010). Synlett.

[cit16] Verho O., Baeckvall J.-E. (2015). J. Am. Chem. Soc..

[cit17] Sun Z., Lonsdale R., Ilie A., Li G., Zhou J., Reetz M. T. (2016). Catalysis.

[cit18] Köhler V., Turner N. J. (2015). Chem. Commun..

[cit19] Turner N. J. (2010). Curr. Opin. Chem. Biol..

[cit20] Díaz-Ro-dríguez A., Lavandera I., Gotor V. (2015). Curr. Green Chem..

[cit21] Kohler V., Turner N. J. (2015). Chem. Commun..

[cit22] Gröger H., Hummel W. (2014). Curr. Opin. Chem. Biol..

[cit23] Denard C. A., Hartwig J. F., Zhao H. (2013). ACS Catal..

[cit24] Schmidt S., Castiglione K., Kourist R. (2018). Chem.–Eur. J..

[cit25] Yu H., Chen X. (2016). Org. Biomol. Chem..

[cit26] Mendez-Sanchez D., Lavandera I., Gotor V., Gotor-Fernandez V. (2017). Org. Biomol. Chem..

[cit27] Torres S. Y., Brieva R., Rebolledo F. (2017). Org. Biomol. Chem..

[cit28] Pamies O., Backvall J.-E. (2004). Trends Biotechnol..

[cit29] Lee J. H., Han K., Kim M.-J., Park J. (2010). Eur. J. Org. Chem..

[cit30] Azerad R., Buisson D. (2000). Curr. Opin. Biotechnol..

[cit31] Didier E., Loubinoux B., Ramos Tombo G. M., Rihs G. (1991). Tetrahedron.

[cit32] Koesoema A., Standley D. M., Senda T., Matsuda T. (2020). Appl. Microbiol. Biotechnol..

[cit33] Hess M., Antranikian G. (2008). Appl. Microbiol. Biotechnol..

[cit34] Dordick J. S. , Biocatalysts for Industry, Springer, Boston, MA, 1991

[cit35] An J., Nie Y., Xu Y. (2019). Crit. Rev. Biotechnol..

[cit36] Mendez-Sanchez D., Mangas-Sanchez J., Lavandera I., Gotor V., Gotor-Fernandez V. (2015). ChemCatChem.

[cit37] Sato H., Hummel W., Gröger H. (2015). Angew. Chem., Int. Ed..

[cit38] Uthoff F., Sato H., Gröger H. (2017). ChemCatChem.

[cit39] Schnapperelle I., Hummel W., Gröger H. (2012). Chem.–Eur. J..

[cit40] Bradshaw C. W., Hummel W., Womg C. H. (1992). J. Org. Chem..

[cit41] Shaabani A., Mirzaei P., Naderi S., Lee D. G. (2004). Tetrahedron.

[cit42] Miller II A. L., Bowden N. B. (2009). J. Org. Chem..

[cit43] Kagaya S., Araki Y., Hasegawa K. (2000). Chem. Lett..

[cit44] Kagaya S., Sagisaka T., Miwa S., Morioka K., Hasegawa K. (2006). Bull. Chem. Soc. Jpn..

[cit45] Ohno A., Shio T., Yamamoto H., Oka S. (1981). J. Am. Chem. Soc..

[cit46] Yasui S., Ohno A. (1986). Bioorg. Chem..

[cit47] Que L., Tolman W. B. (2008). Nature.

[cit48] Abazid A. H., Clamor N., Nachtsheim B. J. (2020). ACS Catal..

[cit49] Betori R. C., May C. M., Scheidt K. A. (2019). Angew. Chem., Int. Ed..

