# Supplementary material for: Deracemization of 1-phenylethanols in a one-pot process combining Mn-driven oxidation with enzymatic reduction utilizing a compartmentalization technique
Source: RSC Adv. 2022 Apr 6;12(17):10619–24. doi: 10.1039/d2ra01326f (PMC8985327; doi:10.1039/d2ra01326f)
Supplement: RA-012-D2RA01326F-s001 [file RA-012-D2RA01326F-s001.pdf]

Electronic Supplementary Material (ESI) for RSC Advances.
This journal is © The Royal Society of Chemistry 2022

Electronic Supplementary information (ESI) for

Deracemization of 1-Phenylethanols in a One-pot Process Combined Mn-driven Oxidation with Enzymatic Reduction Utilizing Compartmentalization Technique

Hirofumi Sato[1]*; Rei Yamada[2]; Yomi Watanabe[1]; Takaaki Kiryu[1]; Shintaro Kawano[1]; Motohiro Shizuma[1]; Hideya Kawasaki[2]

[1]Osaka Research Institute of Industrial Science and Technology, 1-6-50 Morinomiya, Joto-ku, Osaka 536-8553, Japan

[2]Kansai University, 3-3-35, Yamatecho, Suita, Osaka 564-8680, Japan

List of contents

1. General procedure for the 1-phenylethanol substrate scope
2. General procedure for the synthesis of racemic 1-phenylethanols *rac*-1
3. NMR spectra and chiral HPLC charts of chiral phenylethanols
   Rac-1-(2-chlorophenyl)ethanol
   Rac-1-(3-chlorophenyl)ethanol
   Rac-1-(4-chlorophenyl)ethanol
   Rac-1-(2-methylphenyl)ethanol
   Rac-1-(3-methylphenyl)ethanol
   Rac-1-(4-methylphenyl)ethanol
4. Figures
   Figure S1 The effect of Mn oxidizer on the enzyme activity.
   Figure S2 Mn isolation in the interior of PDMS thimble
   Figure S3 ^{1}H-NMR chart of Mn oxidation product.
   Figure S4 ^{1}H-NMR chart of expected byproducts

5. HPLC Charts of products shown in Table 5 (the main manuscript)
   2-Cl (**1b**)
   3-Cl (**1c**)
   4-Cl (**1d**)

2-Me (**1e**)

3-Me (**1f**)

4-Me (**1g**)

1. General procedure for the 1-phenylethanol substrate scope

Magnetic stirrer bar was placed in a PDMS thimble, and then the PDMS thimble was placed in a round-bottomed flask. Dichloromethane solution of substituted *rac*-1-phenylethanol (***rac*-1**, 1.00 mmol in 4.0 mL) was added into the inside of PDMS thimble, and then Mn oxidant was also added to the inside of the PDMS thimble. The mixture was stirred for 24 hours at 25 °C. After the completion of the oxidation, *i*-PrOH (15 mL), phosphate buffer solution (15 mL, 50 mM, pH 7.0), and another magnetic stirrer bar were added to the exterior of the PDMS thimble. Then, $NADP^+$ (15 mg, 20 μmol) and ADH from *Lactobacillus kefir* (*LK*-ADH, 500 U/mmol, 1400 U/mL) were added to the exterior and the reaction mixture was stirred for 24 hours at 25 °C. After the reaction, the PDMS thimble was removed from the flask, and then the aqueous phase in the flask was subsequently extracted with chloroform (10 mL × 3), the combined extracts were dried over sodium sulfate and concentrated in vacuum. To quantify and qualify the product in the interior the removed PDMS thimble with Mn oxidant debris was also washed with chloroform, and then filtered. The filtrate was concentrated in vacuum. The resulting products both from the interior and the exterior were quantified by ^1H-NMR with *t*-BuOH as an internal standard. The enantiomeric excess was evaluated by chiral HPLC.

2. General procedure for the synthesis of racemic 1-phenylethanols *rac*-1

The corresponding acetophenone (5 mmol) was dissolved in 10 mL of methanol. Subsequently, sodium borohydride (7.53 mmol) was added at 0 °C and the solution was stirred over night at room temperature. After completion of the reaction, 10 mL of saturated ammonium chloride solution was added to the mixture. The desired compound was extracted with methylene chloride (3 x 10 mL) and the organic layer was evaporated to concentrate. The residue was purified by silica gel column chromatography to obtain the desired racemic (substituted) 1-phenylethanol.

3. NMR spectra and chiral HPLC charts of chiral phenylethanols

***Rac*-1-(2-chlorophenyl)ethanol**

According to the general procedure, 2-chloroacetophenone was used to yield the product *rac*-1-(2-chlorophenyl)ethanol as colorless oil (619 mg, 3.9 mmol, 79%).

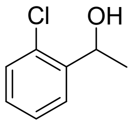

Chemical Formula: C_8H_9ClO

Molecular Weight: 156.61

^{1}H NMR

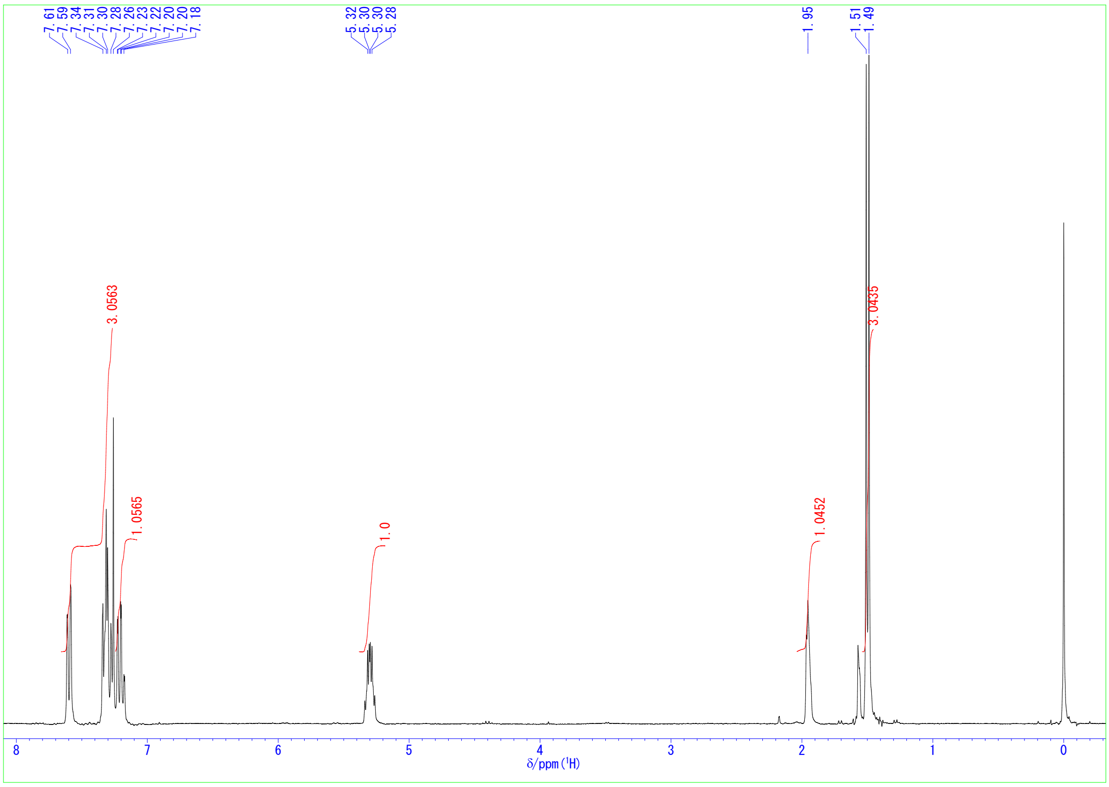

^{13}C NMR

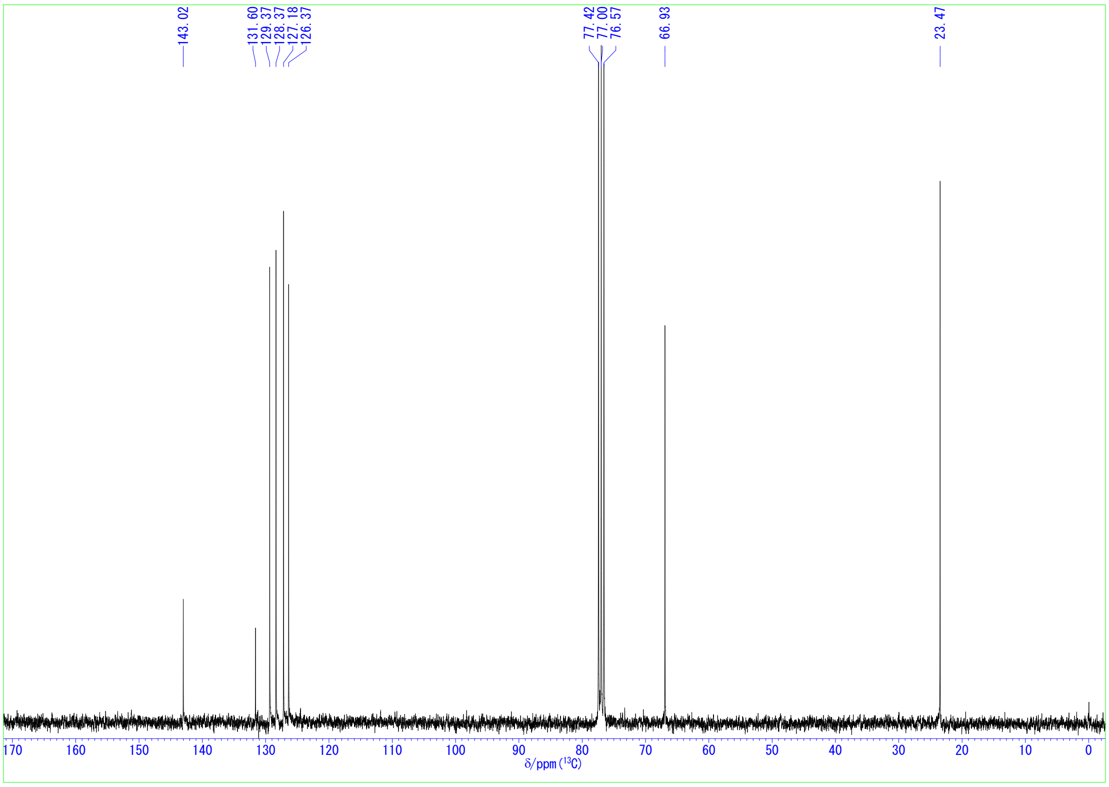

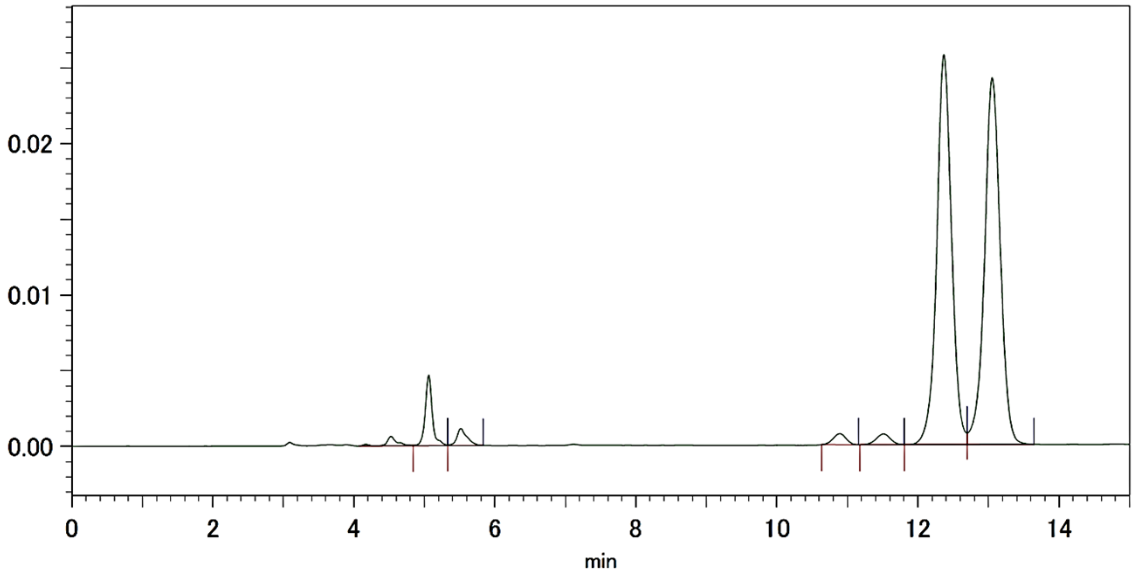

HPLC (YMC CHIRAL Cellulose-C 250 x 4.6 mmI.D. S-5 µm, 1.5%IPA/hexane, flow 1.0 mL/min, 254 nm, 40 °C): $t_{R(S)}$ 12.4 min, $t_{R(R)}$ 13.1 min.

Rac-1-(3-chlorophenyl)ethanol

According to the general procedure, 3-chloroacetophenone was used to yield the product *rac*-1-(3-chlorophenyl)ethanol as colorless oil (626 mg, 4.0 mmol, 80%).

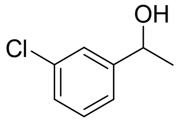

Chemical Formula: C_8H_9ClO

Molecular Weight: 156.61

^{1}H NMR

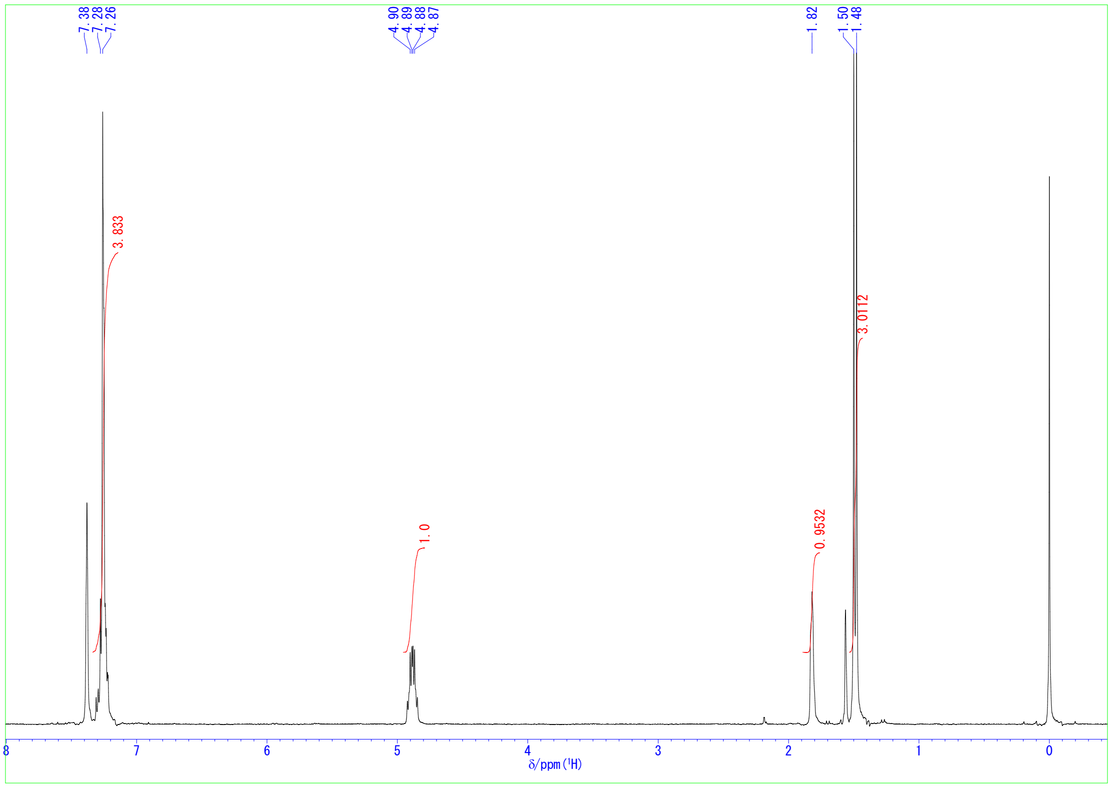

^{13}C NMR

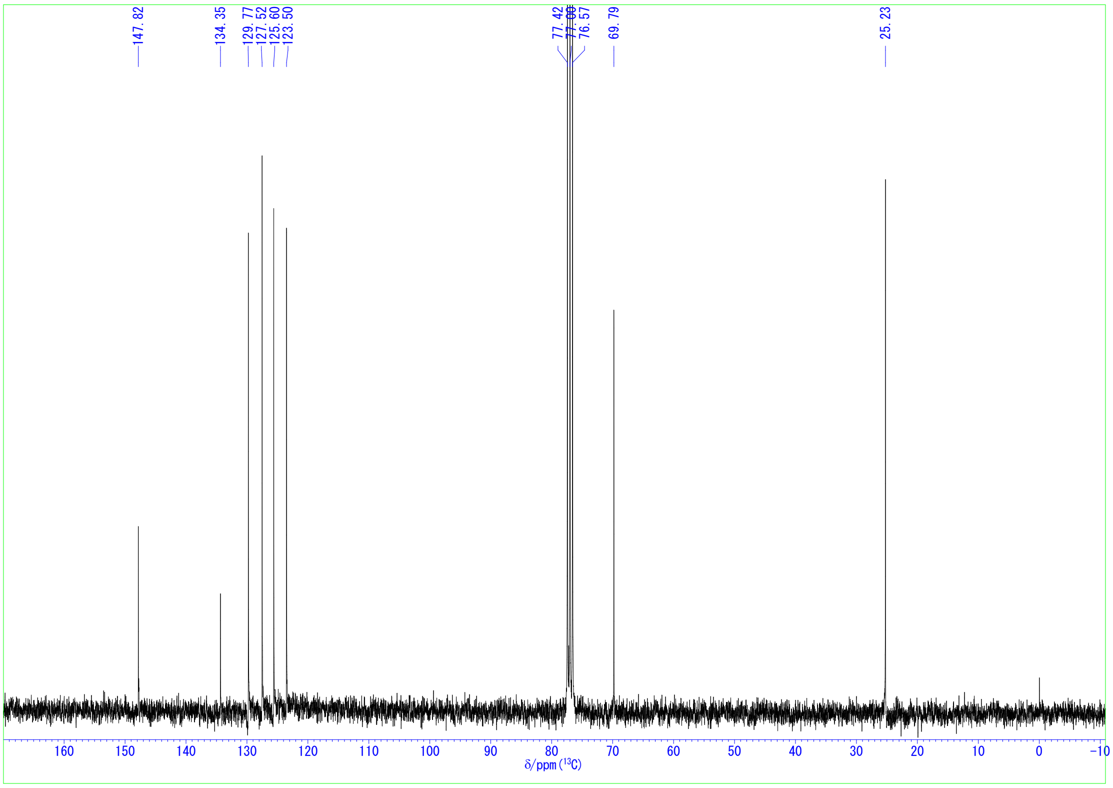

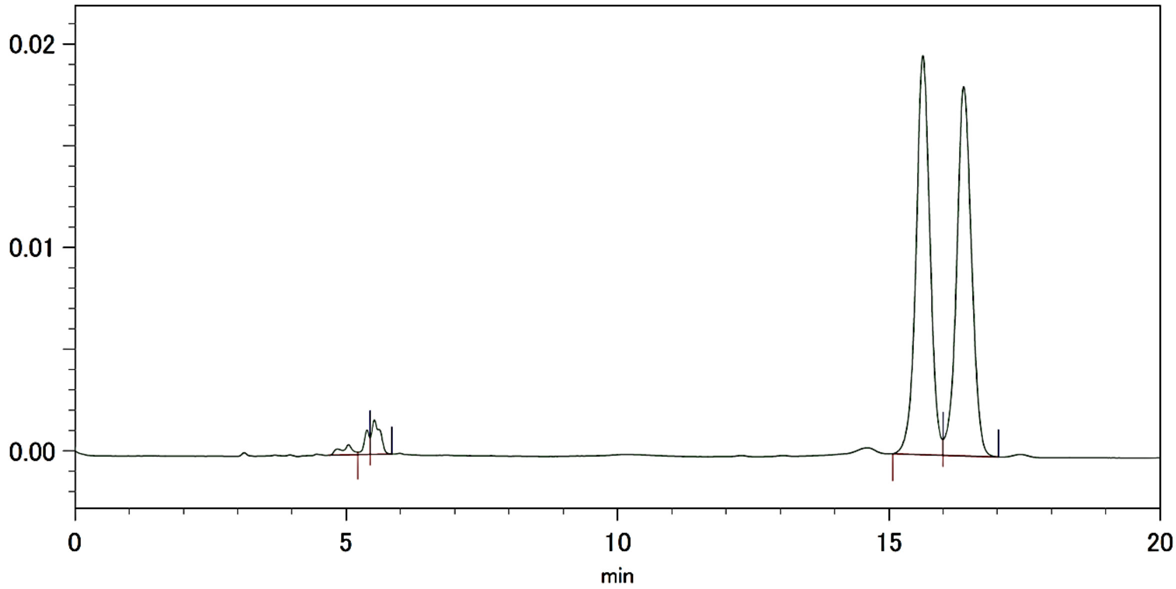

HPLC (YMC CHIRAL Cellulose-C 250 x 4.6 mmI.D. S-5 μm, 1.5%IPA/hexane, flow 1.0 mL/min, 254 nm, 40 °C): $t_{R(S)}$ 15.6 min, $t_{R(R)}$ 16.4 min.

***Rac*-1-(4-chlorophenyl)ethanol**

According to the general procedure, 4-chloroacetophenone was used to yield the product *rac*-1-(4-chlorophenyl)ethanol as colorless oil (665 mg, 4.2 mmol, 85%).

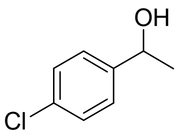

Chemical Formula: C_8H_9ClO

Molecular Weight: 156.61

^{1}H NMR

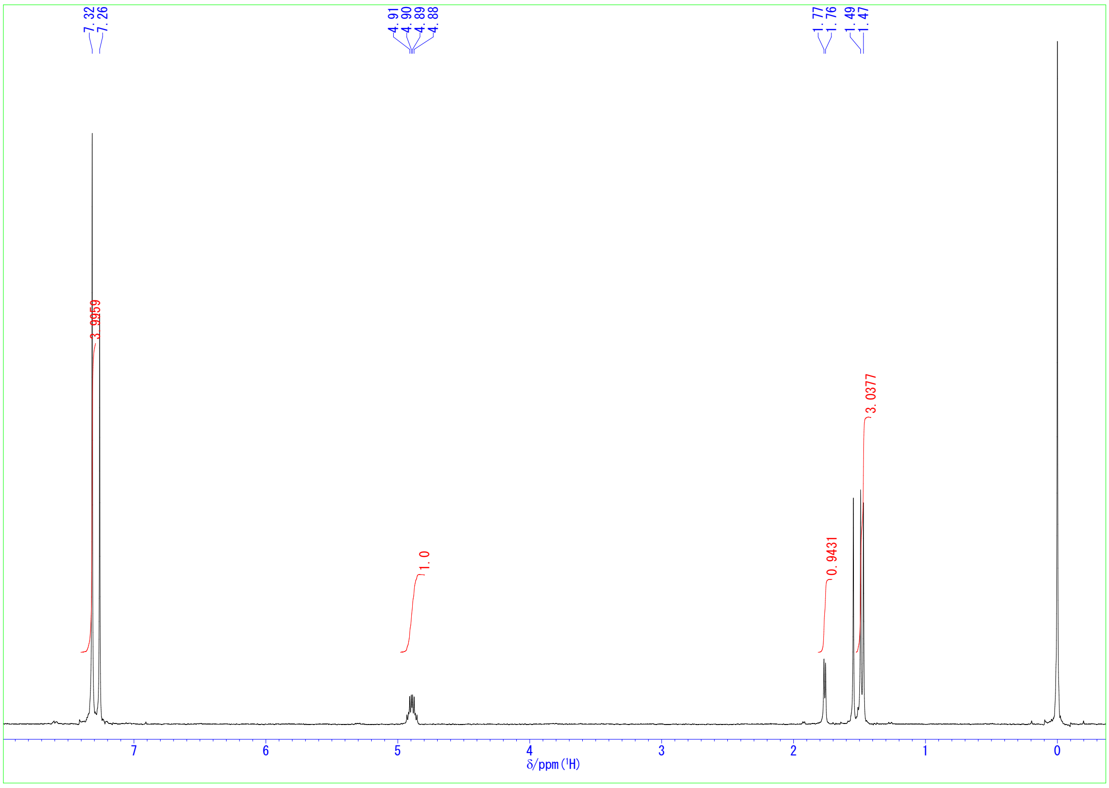

^{13}C NMR

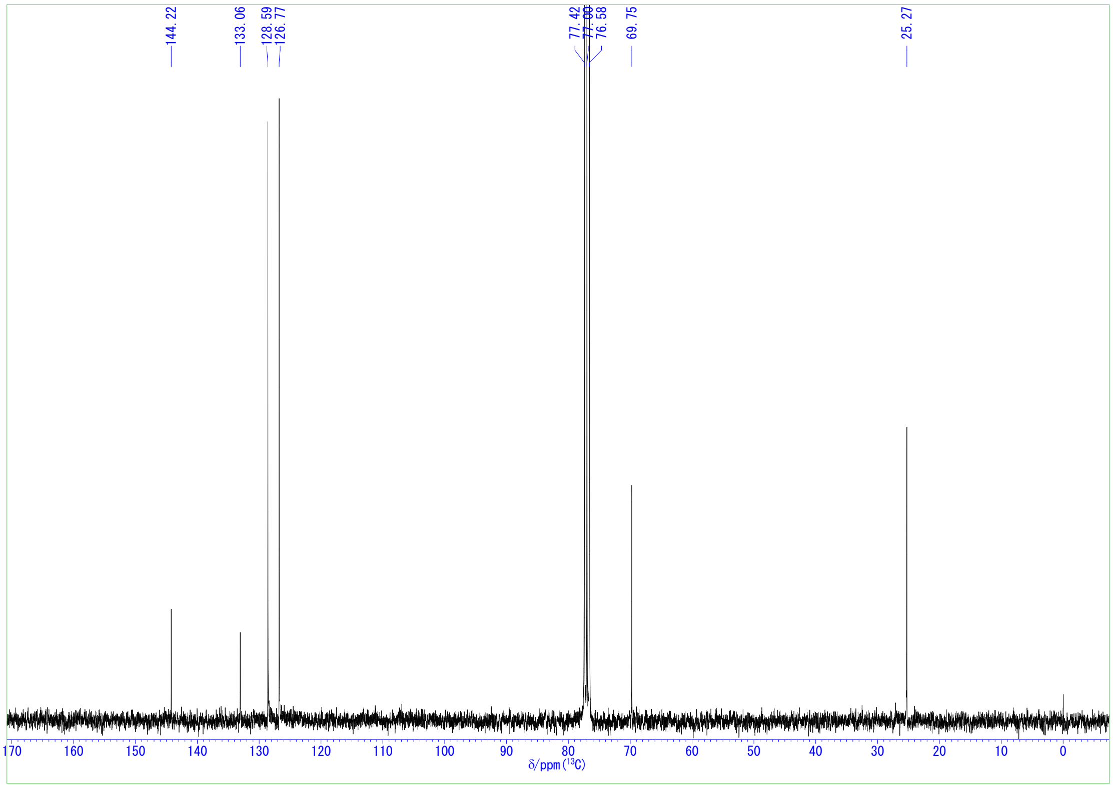

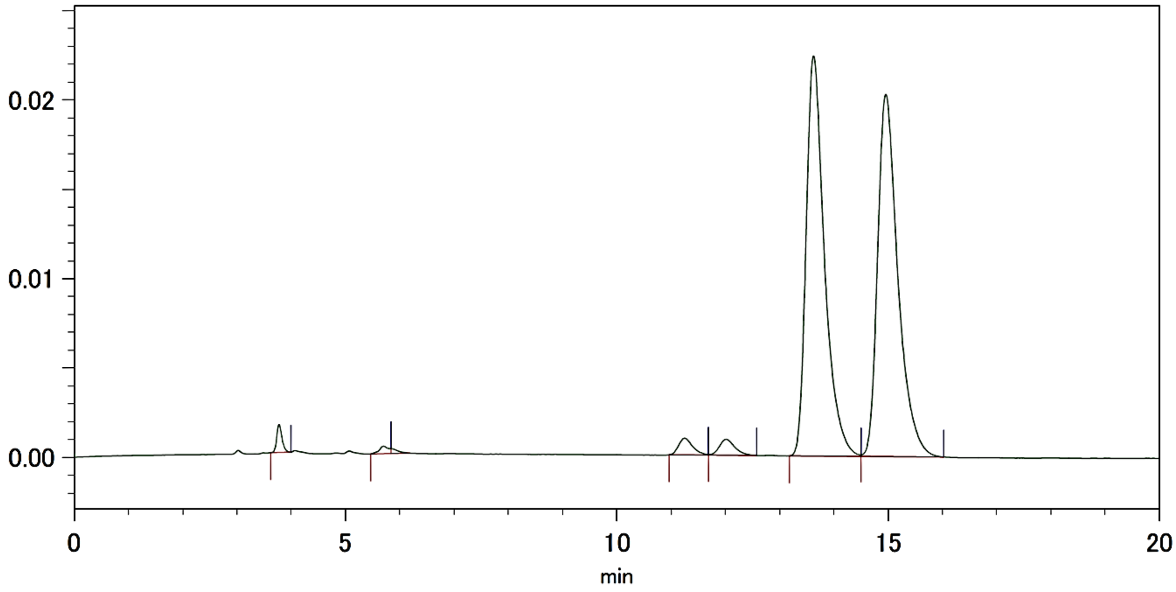

HPLC (DAICEL CHEMICAL IND., LTD. CHIRALCEL OD 250 x 4.6 mmI.D. S-10 μm, 2%IPA/hexane, flow 1.0 mL/min, 254 nm, 40 °C): $t_{R(S)}$ 13.6 min, $t_{R(R)}$ 14.9 min.

***Rac*-1-(2-methylphenyl)ethanol**

According to the general procedure, 2-methylacetophenone was used to yield the product *rac*-1-(2-methylphenyl)ethanol as colorless oil (538 mg, 3.9 mmol, 79%).

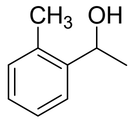

Chemical Formula: $C_9H_{12}O$

Molecular Weight: 136.19

^{1}H NMR

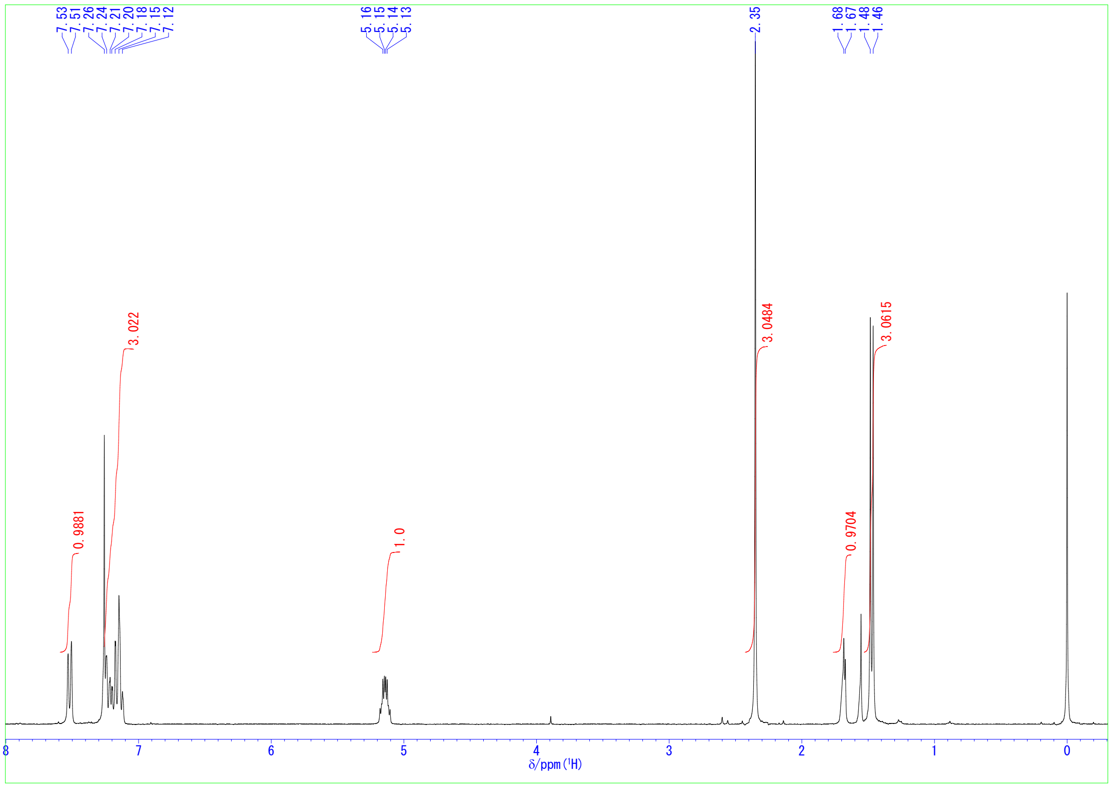

^{13}C NMR

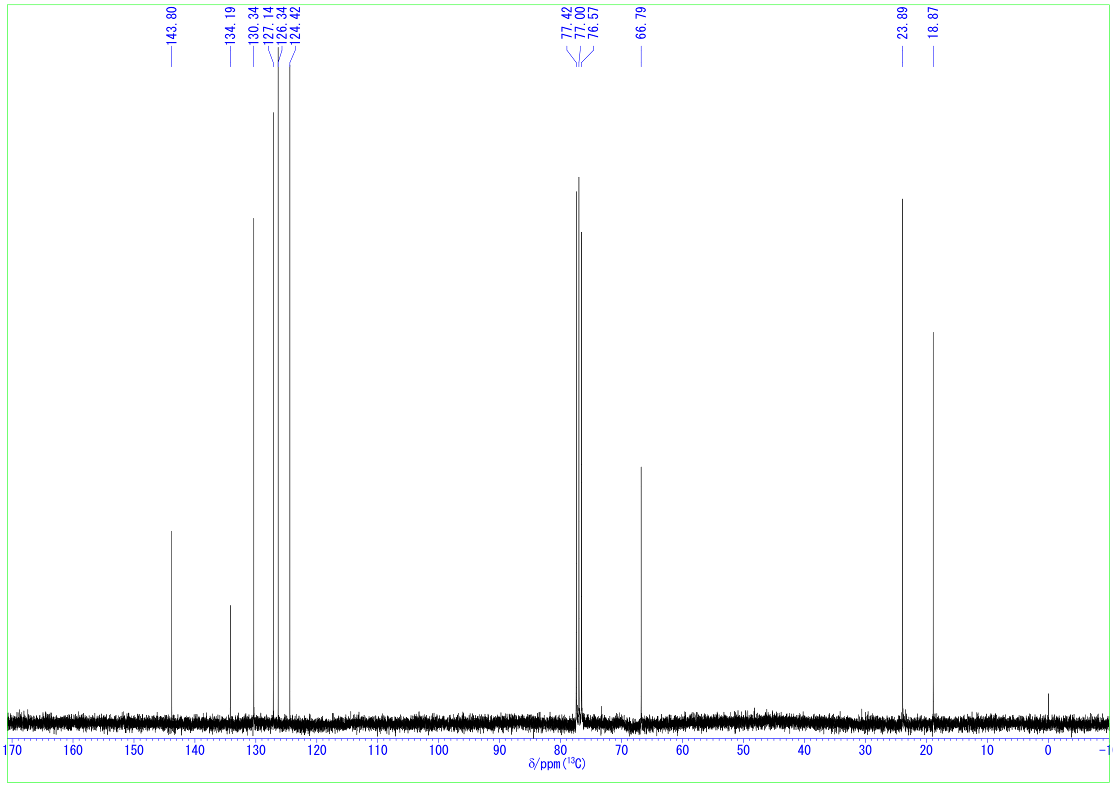

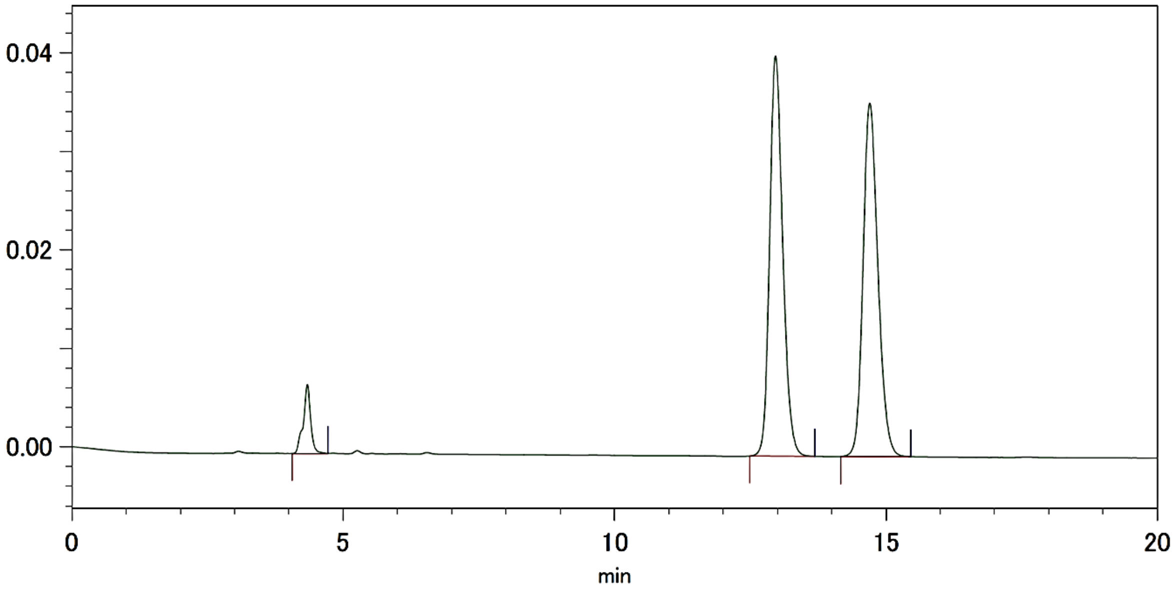

HPLC (YMC CHIRAL Amylose-C 250 x 4.6 mmI.D. S-5 µm, 1.5%IPA/hexane, flow 1.0 mL/min, 254 nm, 40 °C): $t_{R(S)}$ 12.9 min, $t_{R(R)}$ 14.7 min.

Rac-1-(3-methylphenyl)ethanol

According to the general procedure, 3-methylacetophenone was used to yield the product *rac*-1-(3-methylphenyl)ethanol as colorless oil (538 mg, 3.9 mmol, 79%).

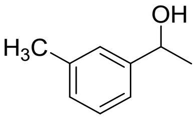

Chemical Formula: $C_9H_{12}O$

Molecular Weight: 136.19

^1H NMR

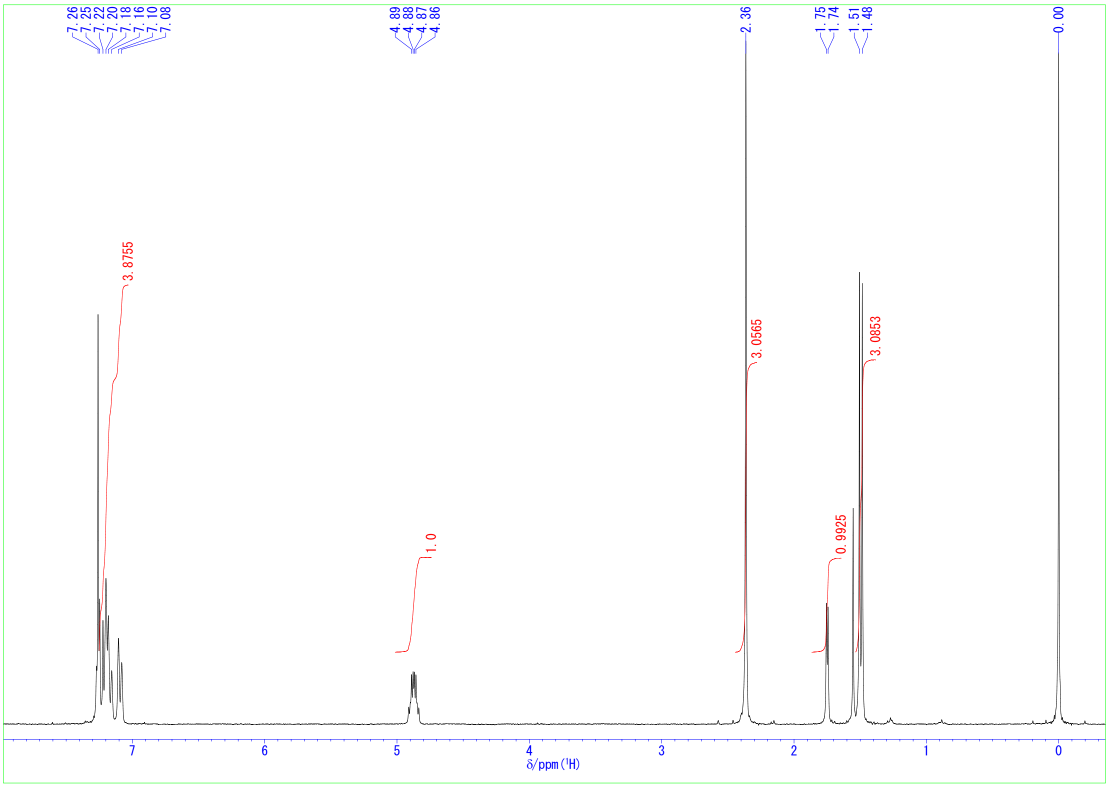

^{13}C NMR

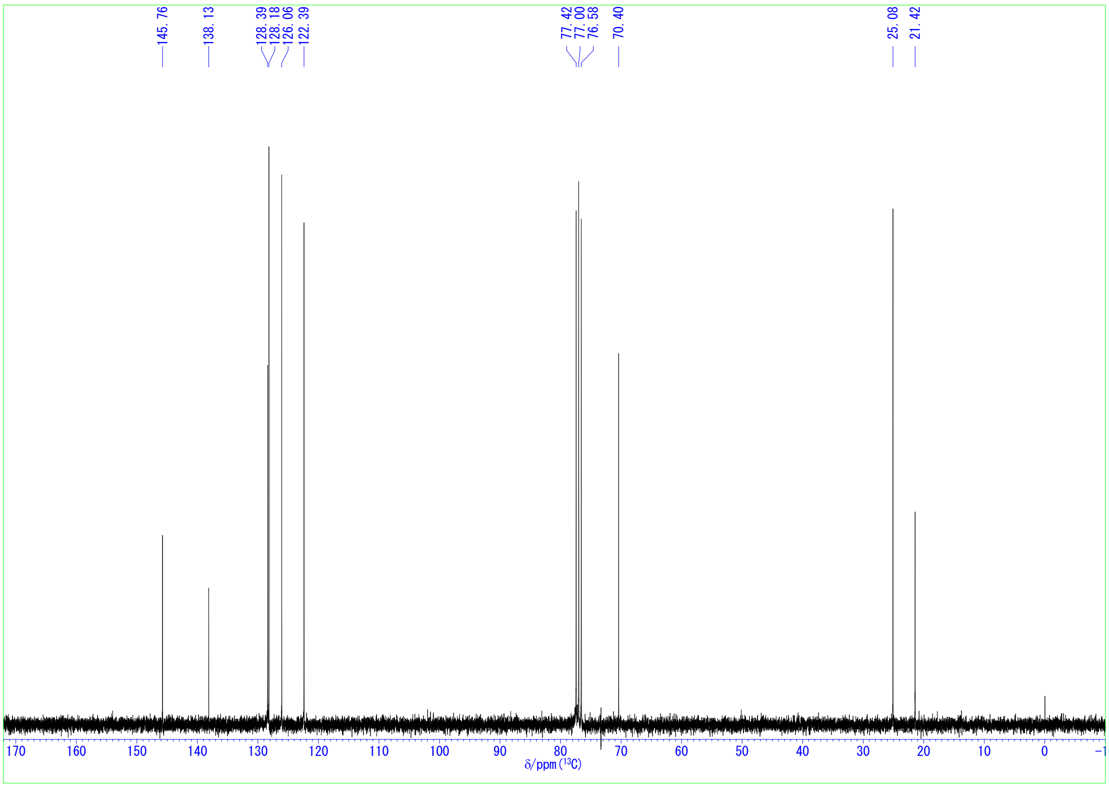

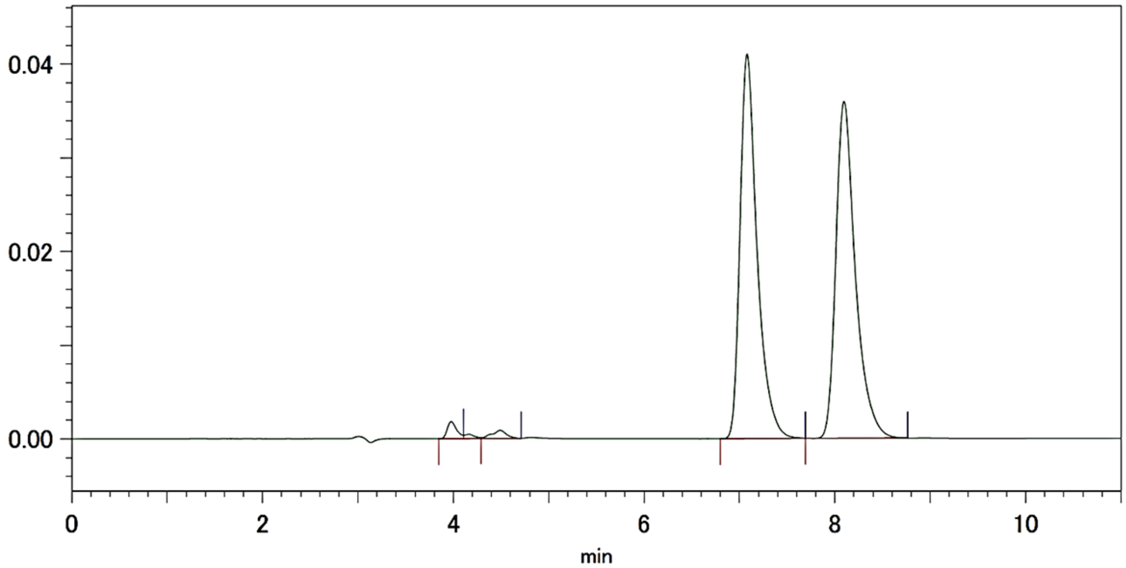

HPLC (DAICEL CHEMICAL IND., LTD. CHIRALCEL OD 250 x 4.6 mmI.D. S-10 μm, 5%IPA/hexane, flow 1.0 mL/min, 254 nm, 40 °C): $t_{R(S)}$ 7.1 min, $t_{R(R)}$ 8.1 min.

Rac-1-(4-methylphenyl)ethanol

According to the general procedure, 3-methylacetophenone was used to yield the product *rac*-1-(3-methylphenyl)ethanol as colorless oil (538 mg, 3.9 mmol, 79%).

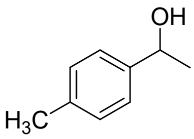

Chemical Formula: $C_9H_{12}O$

Molecular Weight: 136.19

^{1}H NMR

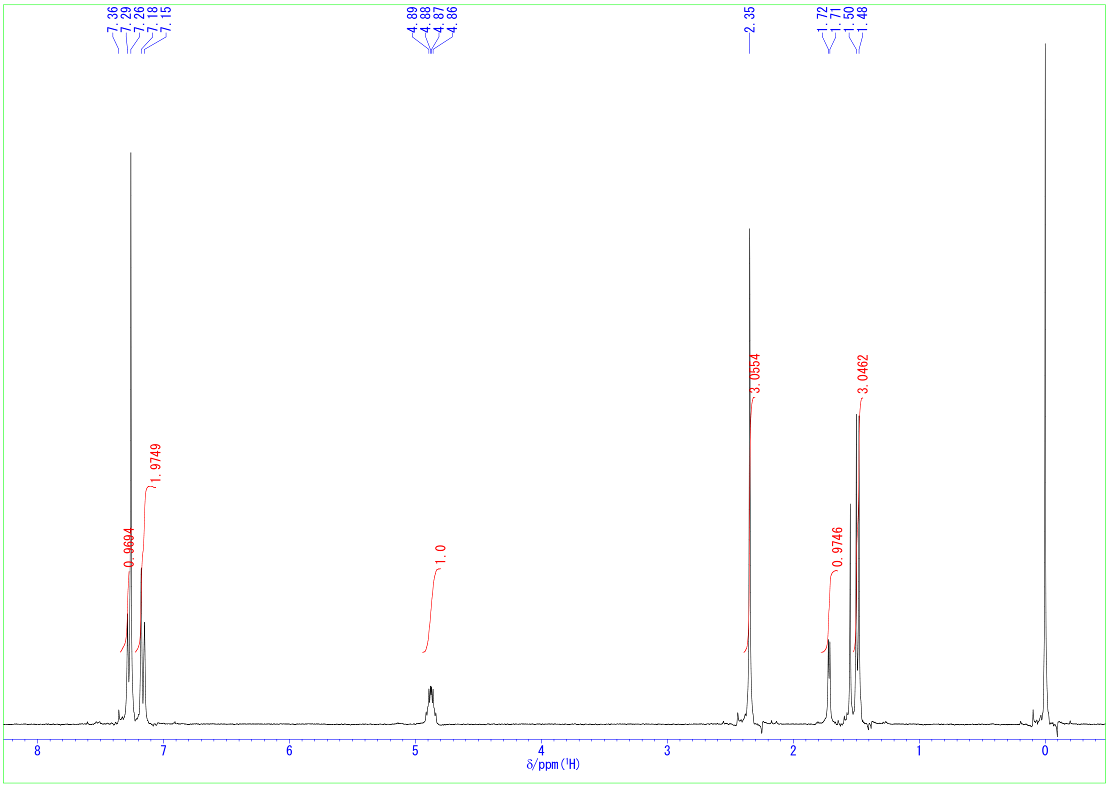

^{13}C NMR

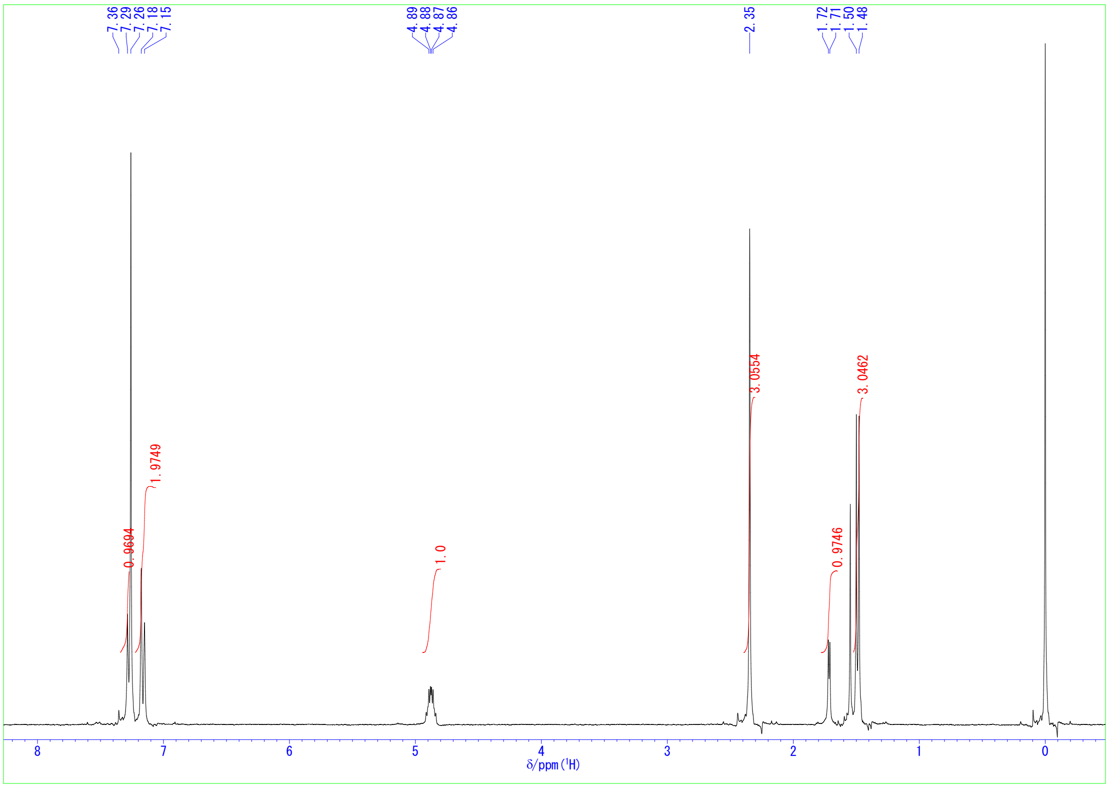

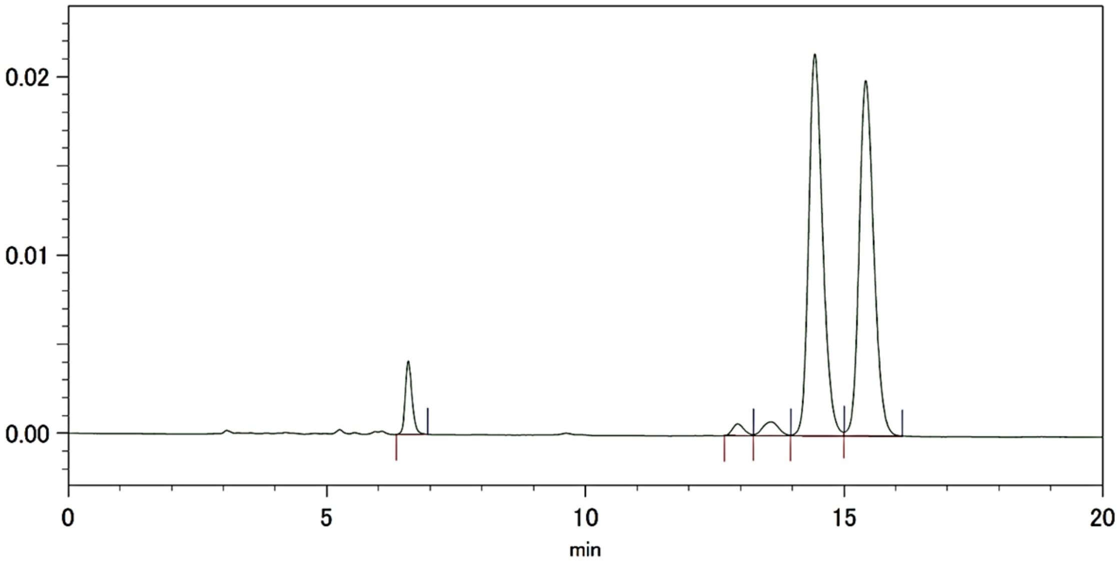

HPLC (YMC CHIRAL Amylose-C 250 x 4.6 mmI.D. S-5 μm, 5%IPA/hexane, flow 1.0 mL/min, 254 nm, 40 °C): $t_{R(S)}$ 14.2 min, $t_{R(R)}$ 15.2 min.

4. Figures

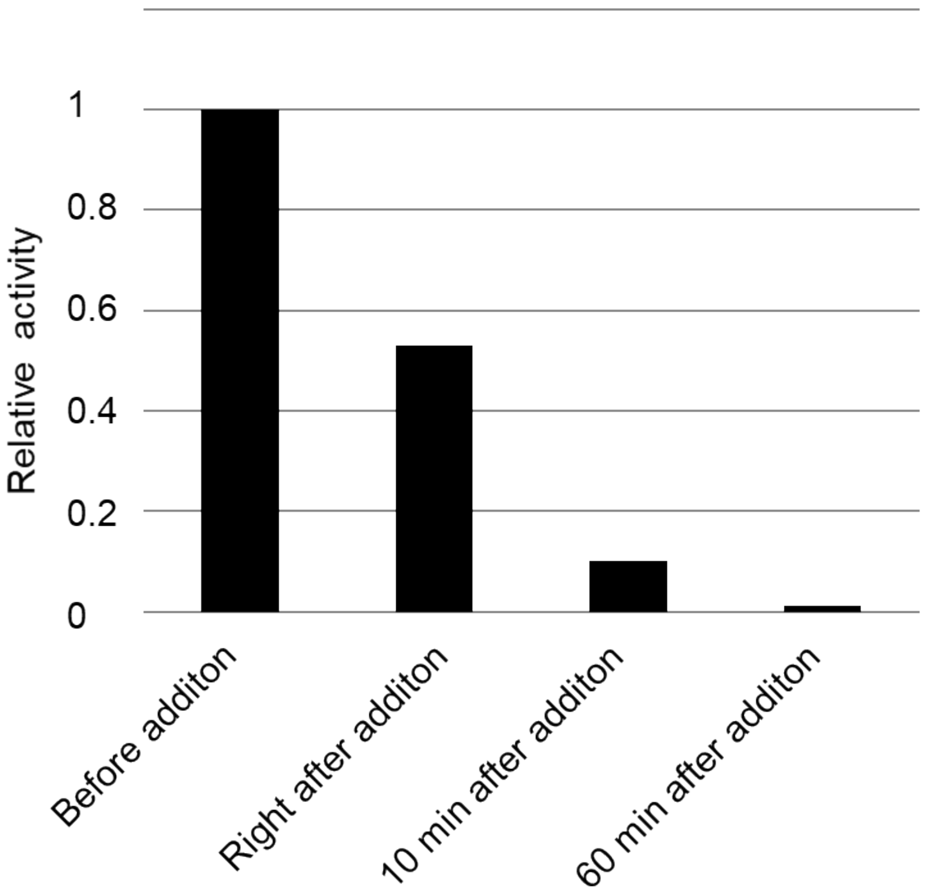

Figure S1 The effect of Mn oxidizer on the enzyme activity.

4-1. Barrier property of PDMS thimble

To prevent the deactivation of exterior enzyme by Mn-oxide, the permeability of Mn species through PDMS thimble was evaluated by monitoring Mn species in exterior chamber. Suspension of Mn oxidant in DCM was poured into the interior of PDMS thimble, and the exterior was filled with IPA/water. After 24 hours, the concentration of Mn species in the exterior was evaluated by ICP-AES. The detected concentration was below 100 ppb at any amount of Mn oxidant from 0.5 g to 3 g (Figure S2); there was no correlation between the added and the detected level. Mn species were turned out to be perfectly isolated by PDMS membrane as the same extent was detected from the blank sample.

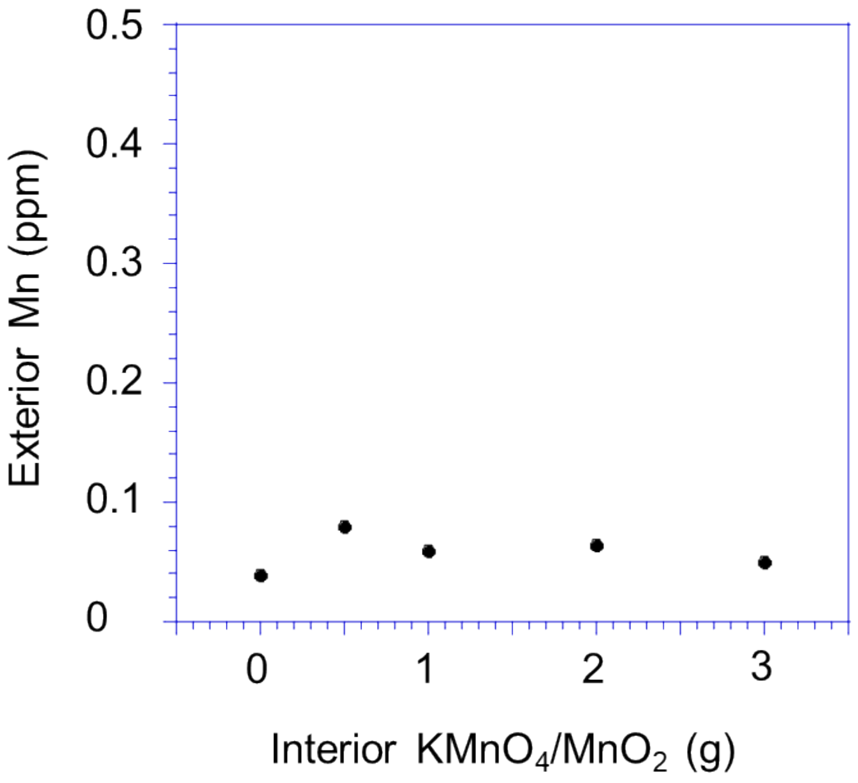

Figure S2 Mn isolation in the interior of PDMS thimble.

4-2 Detailed Mn oxidant preparation and the oxidation product

$KMnO_4$ and MnO_2 (1/3 w/w, for example 0.5 g and 1.5 g, respectively) was ground with agate mortar for up to 5 min until they became powder. The mortar was placed in an oven and dried at 210 °C for 24 hours. After cool in desiccator, the Mn oxidant was used for the oxidation.

Racemic 1-phenylethanol was oxidized, and the product was evaluated by ^{1}H-NMR. In the reaction, only acetophenone was formed and no aldehydes or carboxylic acids (8-12 ppm) were observed (Figure S3). NMR chart of the expected byproducts (products of Wacker oxidation) is shown in Figure S4 as a reference.

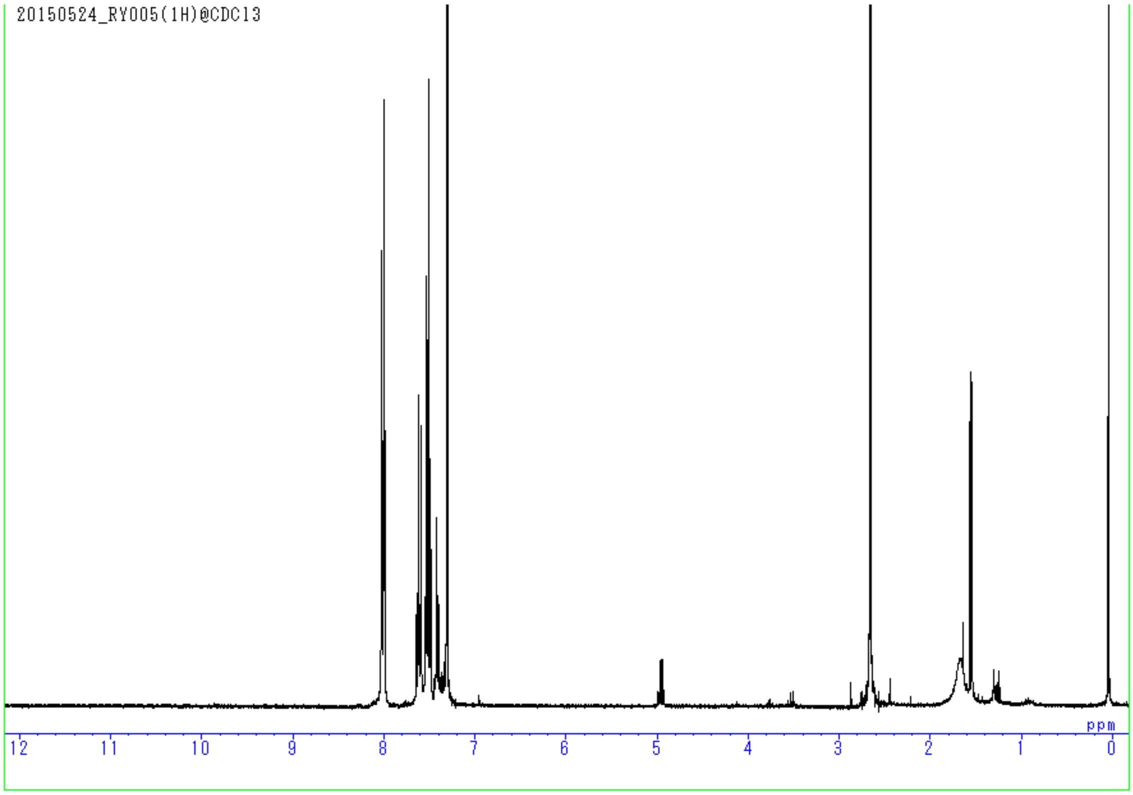

Figure S3 ^{1}H NMR chart of Mn oxidation product.

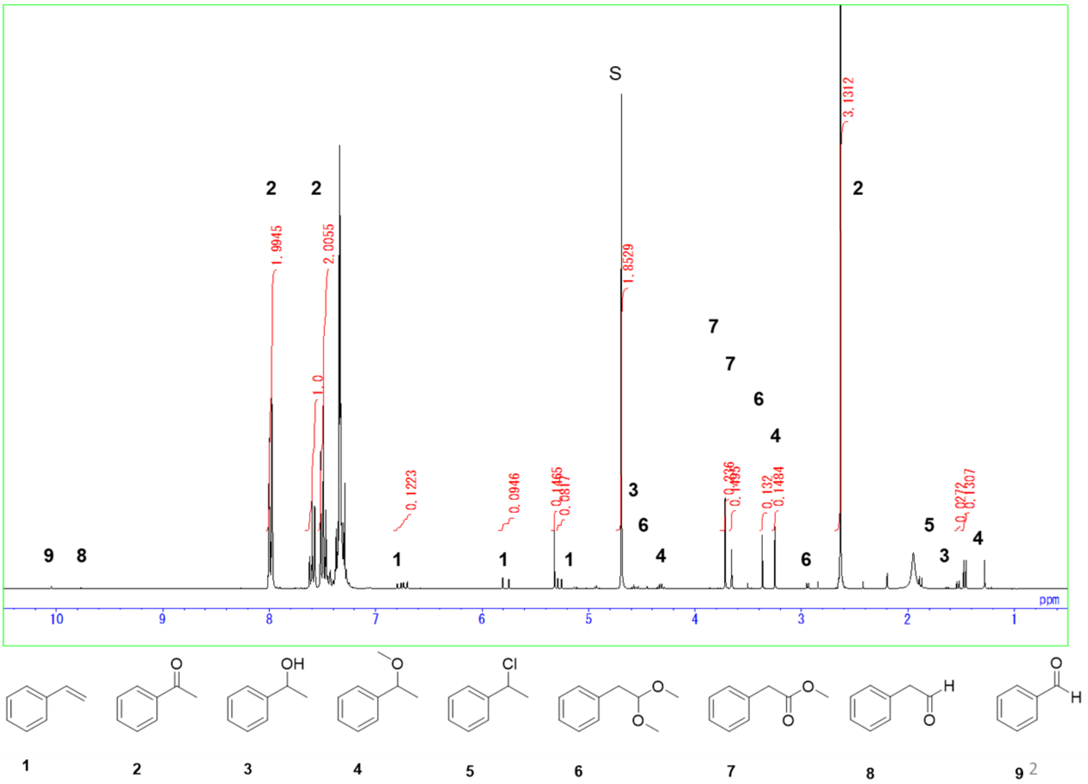

Figure S4 ^{1}H NMR chart of expected byproducts.

5. HPLC Charts of products shown in Table 5 (main manuscript)

2-Cl (1b)

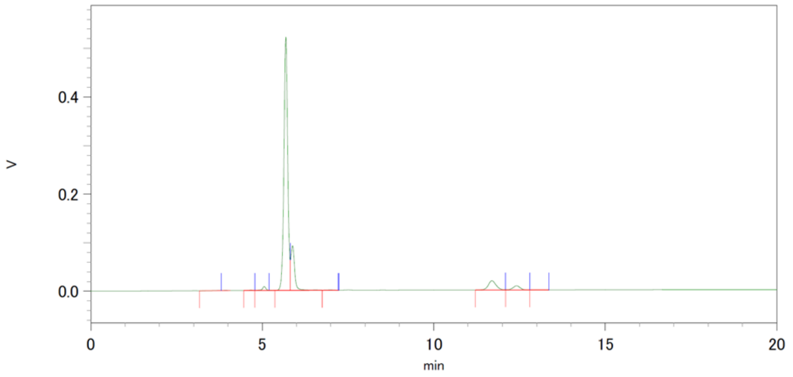

Detector A (254 nm)

Retention time (min)	Area	Area%	Height	Height%
3.583	8979	0.17	328	0.05
4.662	5796	0.11	765	0.12
5.052	56820	1.09	8104	1.24
5.681	4003094	76.89	521740	80.09
5.877	687805	13.21	91769	14.09
6.979	5203	0.10	574	0.09
11.689	306750	5.89	19101	2.93
12.409	126978	2.44	8703	1.34
13.092	5032	0.10	327	0.05

HPLC (YMC CHIRAL Cellulose-C 250 x 4.6 mmI.D. S-5 μm, 1.5%IPA/hexane, flow 1.0 mL/min, 254 nm, 40 °C): $t_{R(S)}$ 12.4 min, $t_{R(R)}$ 13.1 min.

3-Cl (1c)

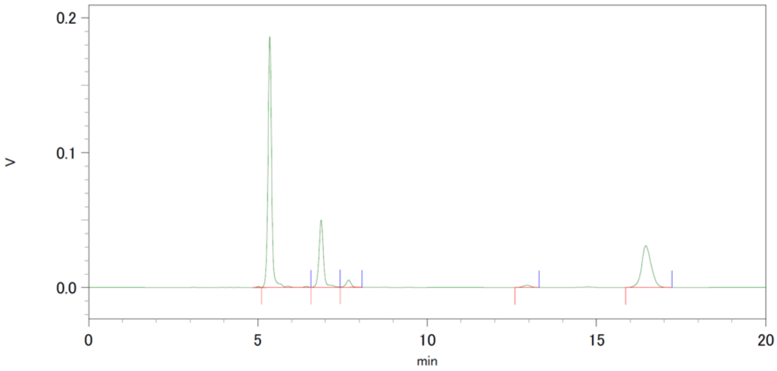

Detector A (254 nm)

Retention time (min)	Area	Area%	Height	Height%
5.347	1384309	54.51	186137	67.89
6.865	457024	18.00	49930	18.21
7.677	56081	2.21	5455	1.99
12.943	25755	1.01	1607	0.59
16.452	616294	24.27	31062	11.33

HPLC (YMC CHIRAL Cellulose-C 250 x 4.6 mmI.D. S-5 µm, 1.5%IPA/hexane, flow 1.0 mL/min, 254 nm, 40 °C): $t_{R(S)}$ 15.6 min, $t_{R(R)}$ 16.4 min.

4-Cl (1d)

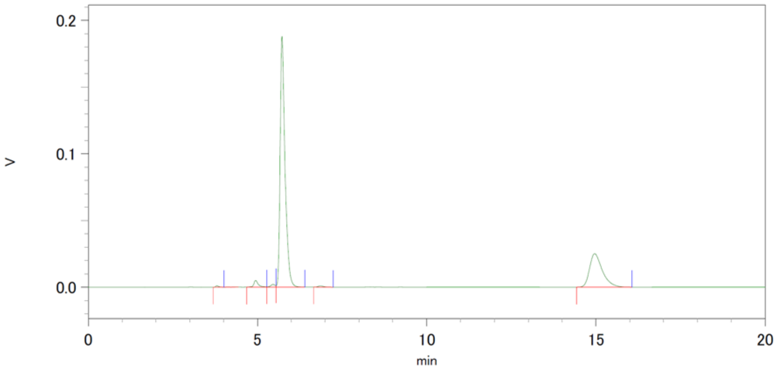

Detector A (254 nm)

Retention time (min)	Area	Area%	Height	Height%
3.798	7139	0.27	1091	0.49
4.939	44648	1.71	4966	2.24
5.452	17405	0.67	2008	0.90
5.719	1875298	71.67	188043	84.66
6.854	10628	0.41	927	0.42
14.959	661341	25.28	25090	11.30

HPLC (DAICEL CHEMICAL IND., LTD. CHIRALCEL OD 250 x 4.6 mmI.D. S-10 µm, 2%IPA/hexane, flow 1.0 mL/min, 254 nm, 40 °C): $t_{R(S)}$ 13.6 min, $t_{R(R)}$ 14.9 min.

2-Me (1e)

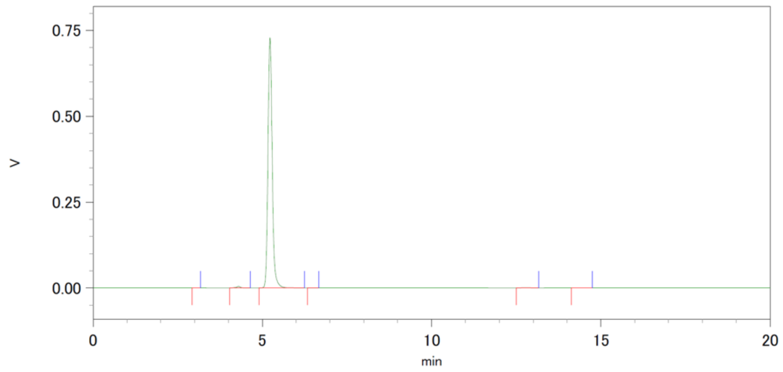

Detector A (254 nm)

Retention time (min)	Area	Area%	Height	Height%
3.040	1181	0.02	215	0.03
4.292	38561	0.60	4392	0.60
5.221	6368173	99.17	728893	99.24
6.461	1215	0.02	150	0.02
12.772	11493	0.18	743	0.10
14.350	915	0.01	54	0.01

HPLC (YMC CHIRAL Amylose-C 250 x 4.6 mmI.D. S-5 µm, 1.5%IPA/hexane, flow 1.0 mL/min, 254 nm, 40 °C): $t_{R(S)}$ 12.9 min, $t_{R(R)}$ 14.7 min.

3-Me (1f)

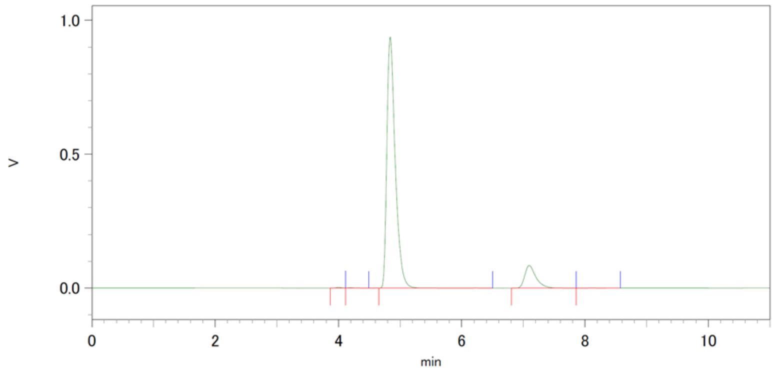

Detector A (254 nm)

Retention time (min)	Area	Area%	Height	Height%
3.996	17930	0.18	2623	0.26
4.193	14301	0.14	1724	0.17
4.835	8829184	88.76	938353	91.27
7.091	1078225	10.84	84894	8.26
8.111	7907	0.08	468	0.05

HPLC (YMC CHIRAL Amylose-C 250 x 4.6 mmI.D. S-5 µm, 5%IPA/hexane, flow 1.0 mL/min, 254 nm, 40 °C): $t_{R(S)}$ 7.1 min, $t_{R(R)}$ 8.1 min.

4-Me (1g)

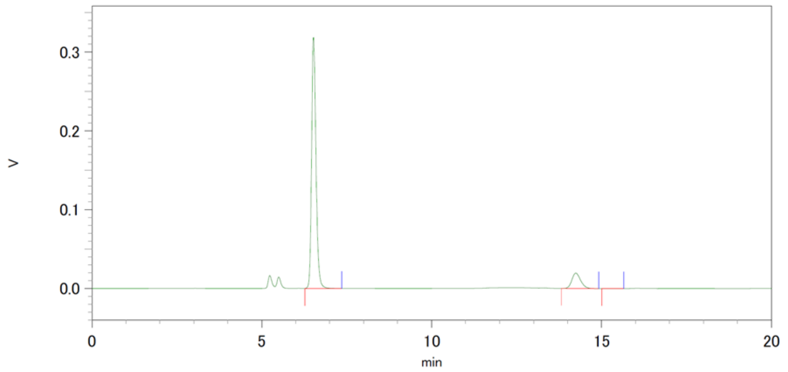

Detector A (254 nm)

Retention time (min)	Area	Area%	Height	Height%
6.517	2850396	89.02	318529	94.19
14.237	350936	10.96	19588	5.79
15.211	595	0.02	43	0.01

HPLC (YMC CHIRAL Amylose-C 250 x 4.6 mmI.D. S-5 µm, 5%IPA/hexane, flow 1.0 mL/min, 254 nm, 40 °C): $t_{R(S)}$ 14.2 min, $t_{R(R)}$ 15.2 min.
